# Taxonomic Novelty and Distinctive Genomic Features of Hot Spring Cyanobacteria

**DOI:** 10.3389/fgene.2020.568223

**Published:** 2020-11-05

**Authors:** Jaime Alcorta, Tomás Alarcón-Schumacher, Oscar Salgado, Beatriz Díez

**Affiliations:** ^1^Department of Molecular Genetics and Microbiology, Biological Sciences Faculty, Pontifical Catholic University of Chile, Santiago, Chile; ^2^Max Planck Institute for Marine Microbiology, Bremen, Germany; ^3^Laboratorio de Bioinformática, Facultad de Educación, Universidad Adventista de Chile, Chillán, Chile; ^4^Center for Climate and Resilience Research (CR)2, University of Chile, Santiago, Chile

**Keywords:** cyanobacteria, hot springs, metagenomes, thermophiles, taxonomy, MAGs

## Abstract

Several cyanobacterial species are dominant primary producers in hot spring microbial mats. To date, hot spring cyanobacterial taxonomy, as well as the evolution of their genomic adaptations to high temperatures, are poorly understood, with genomic information currently available for only a few dominant genera, including *Fischerella* and *Synechococcus*. To address this knowledge gap, the present study expands the genomic landscape of hot spring cyanobacteria and traces the phylum-wide genomic consequences of evolution in high temperature environments. From 21 globally distributed hot spring metagenomes, with temperatures between 32 and 75°C, 57 medium- and high-quality cyanobacterial metagenome-assembled genomes were recovered, representing taxonomic novelty for 1 order, 3 families, 15 genera and 36 species. Comparative genomics of 93 hot spring genomes (including the 57 metagenome-assembled genomes) and 66 non-thermal genomes, showed that the former have smaller genomes and a higher GC content, as well as shorter proteins that are more hydrophilic and basic, when compared to the non-thermal genomes. Additionally, the core accessory orthogroups from the hot spring genomes of some genera had a greater abundance of functional categories, such as inorganic ion metabolism, translation and post-translational modifications. Moreover, hot spring genomes showed increased abundances of inorganic ion transport and amino acid metabolism, as well as less replication and transcription functions in the protein coding sequences. Furthermore, they showed a higher dependence on the CRISPR-Cas defense system against exogenous nucleic acids, and a reduction in secondary metabolism biosynthetic gene clusters. This suggests differences in the cyanobacterial response to environment-specific microbial communities. This phylum-wide study provides new insights into cyanobacterial genomic adaptations to a specific niche where they are dominant, which could be essential to trace bacterial evolution pathways in a warmer world, such as the current global warming scenario.

## Introduction

Cyanobacteria are photosynthetic microorganisms that shaped the earth’s atmosphere during the Great Oxidation Event 2.6 billion years ago (Castenholz et al., 2001; Schirrmeister et al., 2015). They are morphologically diverse and thrive in most environments exposed to light, such as the ocean, lakes, soils, deserts and hot springs (Castenholz et al., 2001). Indeed, they are members of microbial mat communities from non-acidic hot springs, leading primary production and nitrogen fixation (Castenholz, 1969; Ward et al., 1998; Alcamán et al., 2015). Within these thermal microbial mats, which are found across all continents (Castenholz, 1969; Ionescu et al., 2010), the temperature boundary for cyanobacterial survival is approximately 73°C (Cox et al., 2011). In these environments, the temperature gradient shapes the microbial community (Klatt et al., 2011; Mackenzie et al., 2013), with some thermophilic cyanobacteria living at higher temperatures, and thermotolerant or mesophilic members living in the hot spring outflow or borders (Finsinger et al., 2008; Ward et al., 2012).

Over the last two centuries, several hot spring cyanobacterial species have been described (for descriptions, see Komárek, 1999, 2013; Komárek and Anagnostidis, 2005; Ward et al., 2012). Later, phylogenetic analyses revealed a wide biogeographical distribution of unicellular and filamentous thermal members (Papke et al., 2003; Miller et al., 2007; Ionescu et al., 2010; Sciuto and Moro, 2016), demonstrating that the ability to survive at high temperatures is polyphyletic within the phylum (Sanchez-Baracaldo et al., 2005; Uyeda et al., 2016). Nevertheless, the taxonomic information remains incomplete because most known thermophilic strains have only been assigned at the family or genus level, with newly obtained environmental 16S rRNA gene sequences classified based solely on these references (Ionescu et al., 2010; Mackenzie et al., 2013). This, together with the currently persisting conflict in cyanobacterial taxonomy and nomenclature (see Oren and Ventura, 2017, and references therein), prevents the high-resolution description of new cyanobacterial species and genera from environmental samples.

Three major morphological groups of early-described thermophilic cyanobacteria (Schwabe, 1837; Copeland, 1936) have been widely studied. The first group is the unicellular cyanobacteria that diverged and now specializes along the temperature gradient and vertical layers of hot spring microbial mats (Ward et al., 1998, 2006; Olsen et al., 2015). They survive up to the oxygenic photosynthesis temperature limit (Meeks and Castenholz, 1971; Cox et al., 2011), and are most represented by the genera *Synechococcus* and *Thermosynechococcus*, which comprise two very deep branches near the base of the phylum Cyanobacteria (Shih et al., 2013; Dagan et al., 2013). The second group is represented by filamentous non-heterocystous cyanobacteria, which are morphologically diverse as the *Spirulina*, *Leptolyngbya* and *Phormidium* genera (see Copeland, 1936). Some members have the potential to perform both oxygenic and anoxygenic photosynthesis (Momper et al., 2019). Furthermore, they also are globally distributed and dominant in hot springs (Sciuto and Moro, 2016; Yoon et al., 2017). The third and most studied hot spring morphological group is the filamentous heterocystous cyanobacteria, which includes the true-branching species *Fischerella thermalis*. This group has become a high-temperature model for different research topics, such as photosynthesis, nitrogen fixation, multicellularity and biogeography (Alcorta et al. (2019), and references therein).

Genomes from various hot spring strains have shown diverse adaptations to the thermal environment, such as different strategies for phosphate and nitrogen uptake; light responses at different temperatures and depths; and heterocyst envelope composition (Bhaya et al., 2007; Olsen et al., 2015; Alcorta et al., 2018; Sano et al., 2018). Genome reduction is a main evolutionary trend related to the hot spring environment, such as that observed for *Synechococcus* sp. OS-A and OS-B’, and *Thermosynechococcus* (Larsson et al., 2011). The negative correlation of genome size and protein length with increasing temperature is also a major evolutionary trend related to thermophilic bacteria (Sabath et al., 2013), as is differentiated nucleotide content, codon usage, amino acid composition (Vieille and Zeikus, 2001; Singer and Hickey, 2003; Zeldovich et al., 2007) and the prevalence of the CRISPR-Cas system (Weinberger et al., 2012). However, cyanobacterial genomes are not well represented in these studies; thus, the genomic features and functional categories that differentiate thermophilic cyanobacteria are still unknown.

To obtain new information on hot spring cyanobacterial genomic features, metagenomic reconstruction of complete or partial genomes, known as metagenome-assembled genomes (MAGs), was used to uncover an unprecedent diversity. From 21 globally distributed neutral-pH hot spring microbial mats, with temperatures between 32 and 75°C, 57 new cyanobacterial MAGs were obtained, some of which were classified into well-known genera and species, while others represent new taxa at the order to species levels. Comparative genomics corroborates thermophilic features prevalent in other bacteria, and also reveals new trends related to exclusive orthologs, abundances of protein functional categories and adaptative genes involved in the response to the microbial and viral hot spring community. These results highlight various consequences of the ecological speciation process on thermophilic cyanobacterial genomes. However, more studies are now required to reveal the initial colonization mechanism of these organisms to this extreme habitat.

## Materials and Methods

### Study Sites and Sample Collection

Phototrophic microbial mat samples were taken from El Tatio geyser field (Atacama, Chile), Cahuelmó hot spring (Northern Patagonia, Chile) and Kroner Lake (Deception Island, Antarctica). The sample location, collection year and physicochemical parameters are listed in Supplementary Table 1. Temperature and pH were determined using a multiparameter instrument (model 35607-85; Oakton, Des Plaines, IL, United Stattes). For molecular analysis, 2 cm core samples were collected and frozen at −80°C until subsequent procedures. DNA was isolated using a bead-beating protocol with xanthogenate lysis buffer and a phenol–chloroform extraction, according to Alcorta et al. (2018). The quality and quantity of nucleic acids were checked using the Qubit (LifeTechnologies, Carlsbad, CA, United States) and Nanodrop (Thermo Fisher Scientific, Waltham, MA, United States) systems.

### Sequencing and Read Quality Assessment

For metagenomic analysis, DNA samples were sequenced on the Illumina HiSeq platform (Illumina, San Diego, CA, United States) at the Research and Testing Laboratory (Lubbock, TX, United States). Briefly, the DNA was fragmented using the NEBNext dsDNA Fragmentase kit (New England Biolabs, Ipswich, MA, United States), followed by DNA clean up via column purification and library construction with the NEBUltra DNA Library Prep kit for Illumina (New England Biolabs). Methodology from Guajardo-Leiva et al. (2018) was followed for quality filtering using Cutadapt (Martin, 2011) with the parameters: paired-end mode, a perfect match of at least 10 bp (-O 10) against the standard Illumina adaptors, hard clipping of the first five leftmost bases (-u 5), 3′ end trimming for bases with a quality score below 28 (-q 28) and retaining only sequences longer than 30 bp (-m 30).

### Assembly and Metagenomic Binning

*De novo* assemblies of trimmed reads were generated using SPAdes v3.10.1 (Bankevich et al., 2012) with the –meta option. Contigs longer than 1,000 bp were grouped into MAGs using the metaWRAP binning module (Uritskiy et al., 2018), which incorporates the following three binning software: metaBAT 2 v2.12.1 (Kang et al., 2019), MaxBin2 (Wu et al., 2016) and CONCOCT v1.1.0 (Alneberg et al., 2014) with default parameters. Next, the bin_refinement module of metaWRAP (Uritskiy et al., 2018) was used to consolidate results from the three methods using the -c 50 and -x 10 options to obtain bins with over 50% completeness and less than 10% contamination according to the CheckM tool v1.0.18 (Parks et al., 2015), which also uses HMMER v3.2.1^1^ (Eddy, 1998) as a third-party software. The refineM tool (Parks et al., 2017) was used to clean potential contig contamination with different genomic properties (tetranucleotide signature and coverage) via the scaffold_stats, outliers and filter_bin modules. The refineM tool (Parks et al., 2017) was also used to clean potential contamination based on taxonomic assignment with the following modules: call_genes, which uses Prodigal v.2.6.3 (Hyatt et al., 2010); taxon_profile, using the GTDB R80 custom protein database from the Genome Taxonomy Database (GTDB; Parks et al., 2018; available at^2^); and taxon_filter and ssu_erroneous, using the GTDB R80 custom SSU database^2^. The obtained MAGs were reassessed with the CheckM tool v1.0.18 (Parks et al., 2015), their tRNAs were predicted with the ARAGORN webserver (Laslett and Canback, 2004), and their ribosomal subunit sequences were searched with Barrnap v0.9^3^. According to the Genomic Standards Consortium parameters, this information allowed us to classify the MAGs as high-, medium- or low-quality (Bowers et al., 2017). Taxonomic assignment was performed using GTDB-tk v0.3.2 software with version R89 (Chaumeil et al., 2020), which also uses pplacer as a third-party software (Matsen et al., 2010). Genomes belonging to the phylum Cyanobacteria were used for further analyses. The similarity between MAGs was assessed through all-vs-all comparisons of the average nucleotide identity (ANI) using fastANI software (Jain et al., 2018) and the average amino acid identity (AAI) using compareM software^4^. MAGs identified as possible new taxa were grouped according to thresholds of similarity for the ANI and AAI values stated by Konstantinidis et al. (2017).

Additionally, a locally built database of public metagenomes from hot springs was analyzed. SRA files were first downloaded from the NCBI database, and subsequently quality trimmed, assembled, binned and taxonomically classified as described above. The only procedural difference was that the SRR5581334, SRR7905023 and ERR372908 metagenomes were assembled using MEGAHIT 1.2.9 (Li et al., 2015), due to memory requirements. Details of these metagenomes are listed in Supplementary Table 1. High- and medium-quality MAGs assigned to the phylum Cyanobacteria were used for further analyses. All obtained cyanobacterial MAGs were submitted in FASTA format to the Figshare repository^5^ under 10.6084/m9.figshare.12400979. Meanwhile, 34 high- and medium-quality MAGs with > 95% completeness were deposited under NCBI BioProject numbers PRJNA635751 and PRJNA645256. The MAGs obtained using primary data from this study and from Alcamán-Arias et al. (2018) were submitted to the NCBI WGS database, while those obtained from Chan et al. (2015), Kaushal et al. (2018), Ward et al. (2019) and Roy et al. (2020) were submitted to the Third Party Annotation database^6^.

### Abundance of MAGs in Metagenomes

The abundance of the recovered MAGs was assessed through read mapping. Briefly, quality trimmed reads of each sample were mapped using BBMap v38.71^7^ with a minimum identity of 99% (minid = 0.99 and idfilter = 0.97). A MAG was considered present in a sample when it had a coverage > 1x across 75% of its genome; otherwise, the abundance was considered zero. Absolute read counts of selected MAGs were normalized as the number of reads recruited per kilobase of MAG and gigabase of metagenome (RPKG), which allowed the direct comparison of genome abundances between metagenomes of different depths (Reji and Francis, 2020). Normalized read counts were used to calculate diversity metrics, as well as the similarity matrix for multivariate analyses with the “Vegan” package in R.

### Phylogenomics and Taxonomy

All genomes classified as Cyanobacteria (1,626 genomes as of September 2019) were downloaded from the NCBI database, and the GTDB taxonomy was assigned to each using GTDB-tk software (Chaumeil et al., 2020). The dRep v2.3.2 software (Olm et al., 2017) was used to dereplicate the entire set due to overrepresented species and low-quality genomes (e.g., only 1136 exhibited > 75% completeness), thus obtaining a final subset of 800 genomes (Supplementary Table 2). A total of 120 concatenated single-copy bacterial genes were recovered from the intermediate files of the GTDB-tk analysis (gtdbtk.bac120.msa.fasta files), and their sequences were subsequently aligned using MUSCLE v6.0.98 software (Edgar, 2004). Maximum-likelihood trees were generated using IQtree v.1.5.5 software with the TESTNEW option to choose the best substitution models, after which a non-parametric ultrafast bootstrap (-bb) support of 10,000 replicates was applied (Hoang et al., 2017). Node collapse and rooting of phylogenetic reconstructions were managed using the iTOL web server (Letunic and Bork, 2019).

### Comparative Genomics

Genomic features of the 57 MAGs obtained in the present study, as well as for the 800 cyanobacterial genomes from the NCBI database, were extracted from the CheckM summary results (see above, Supplementary Table 2). As the MAGs and NCBI genomes have different completeness levels, the expected genome size was calculated as EGS = (genome size ^∗^ 100)/(completeness). In conjunction with the MAGs, a subset of NCBI genomes that were taxonomically close to the MAGs (according to GTDB-tk), was used to compare the genome size, GC percentage and coding density between hot spring cyanobacteria (all 57 MAGs and 36 NCBI genomes) and non-thermal cyanobacteria (66 NCBI genomes). Together, these 159 genomes are hereafter referred to as the 159-subset. Additionally, the hydrophobicity, protein length, protein molecular weight, isoelectric point (pI) and amino acid usage were compared for the coding DNA sequences (CDSs) calculated with ProPAS v1.03 software (Wu and Zhu, 2012). Furthermore, genomes from both environmental groups were compared for the Thermosynechococcacceae, *Elainellaceae* and Oscillatoriaceae families, and for the *Fischerella* and *Geminocystis* genera (≥ 3 genomes for each environment) to identify differences at these specific taxonomic levels. The isolation environments and associated references for each cyanobacterial genome of the 159-subset are listed in Supplementary Table 3.

Additionally, the 159-subset orthogroups (orthologous gene clusters, see Supplementary Table 4) obtained with Orthofinder v2.3.3 software (Emms and Kelly, 2015), were used to identify the core and accessory orthogroups. Orthogroups present in > 97% of the subset (153 genomes) were considered the core genome (based on their distribution in the total 47,328 obtained orthogroups). Orthogroups sporadically present in both environmental groups were considered the phylum accessory set, while those exclusive to hot spring or non-thermal genomes were considered as the specific accessory orthogroups. This includes the singletons as well as the specific core accessory orthogroups if they have presence in all genomes an environmental group. This same classification was also performed for seven genera with genomes from both environmental groups (*Calothrix*, *Cyanobacterium*, *Elainella*, *Fischerella*, *Geminocystis*, *Rivularia* and *Trichormus*) and a heatmap of the relative abundance of COG categories was created with R software to make a hierarchical clustering (hclust) of the core and accessory categories. Orthogroups distributed in more than one order, family or genus in each environmental group, were further explored by searching for homologous sequences in the NCBI non-redundant protein database using BLASTP (Altschul et al., 1990). From the retrieved sequences, phylogenetic reconstructions were generated as explained above.

Functional annotation of the 159-subset genomes was done with eggnog-mapper software (Huerta-Cepas et al., 2017, 2019). First, the genomic percentage of COG categories in each genome was compared between hot spring and non-thermal cyanobacteria using STAMP v2.1.3 software (Parks et al., 2014) via the Welch’s *t*-test with Bonferroni multiple test correction. Next, specific annotations of complementary metabolism, defense systems and secondary metabolites were searched. Because many proteins are annotated with putative functions or as hypothetical proteins, the following specific terms were used to search within the annotated 159-subset: “nitrogenase,” “nitrate,” “nitrite,” “restriction,” “modification,” “capsid,” “dehydrogenase,” “CRISPR,” “virus,” “viral” and “capsid.” Orthogroups harboring protein sequences whose annotations indeed corresponded to these functions were then used for further analyses. Some orthogroups with ambiguous functions (different annotations within the orthogroup) were ignored. For the sulfide-quinone reductase protein, which is characterized as a possible switch between anoxygenic and oxygenic photosynthesis, BLASTP was used with the reference NCBI sequence (AF242368.1 or WP_071516517.1) to search the corresponding orthogroup. Secondary metabolite biosynthetic gene clusters (BGCs) were searched with antiSMASH v4.2.0 software (Blin et al., 2017) using the clusterblast and borderpredict options.

### Statistical Analyses

Correlation analyses between MAGs abundances and environmental parameters were calculated with the Mantel test implemented in the R package “Vegan,” only considering the variables temperature, pH and geographical location. Additional parameters that were not available for all samples, were excluded from the analyses. Wilcoxon’s paired test was used because the comparison of genome features was not balanced between hot spring and non-thermal genome groups. Correlation analyses were performed by determining the adjustment to the “normal” distribution of each variable with the Kolmogorov-Smirnov test and by using parametric Pearson’s or non-parametric Spearman’s tests, depending on the distribution of data. Correlation indexes were compared using the r-to-z Fischer transformation. For multiple analyses over the same dataset, Bonferroni and FDR corrections were applied to the obtained p-values. The R packages “Tidyverse,” “ggpubr,” “ggplot2,” “cocor,” and “maps” (R Core Team, 2017) were used for all statistical analyses and corrections, as well as most of the plots.

## Results and Discussion

### Hot Spring Cyanobacterial MAG Recovery

**FIGURE 1:**
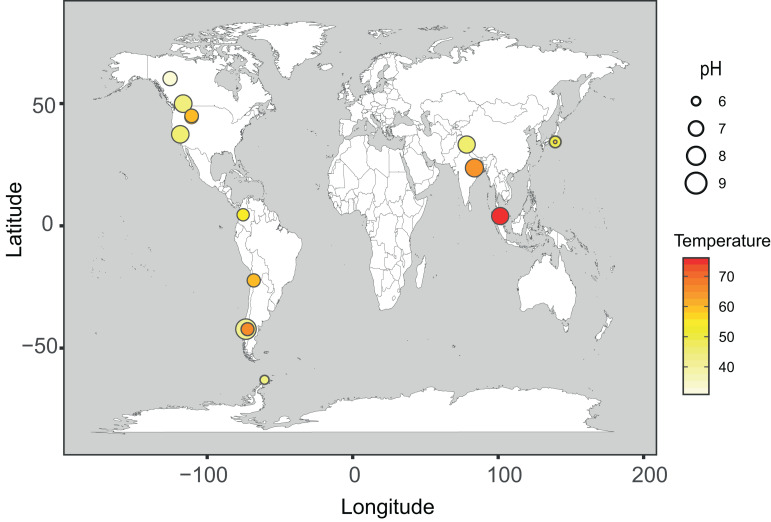
Geographical distribution of phototrophic hot spring metagenomes. Distribution map of the 21 hot spring microbial mat metagenomes used in the present study. Each circle represents one sample. The color scale for circles represents the temperature range from 32 to 75°C, and the circle size represents the pH range (from 5.8 to 9.2) of each sample.

In this study, four new metagenomes from hot spring phototrophic microbial mats in Chile and Antarctica were analyzed along with 79 metagenomes already available in the NCBI database. The four new metagenomes altogether added up to 60.75 Gbp of total trimmed reads and 292,512 assembled contigs (> 1,000 bp), totaling 1.36 Gbp. For the locally built database, cyanobacterial sequences were identified in only 17 of the 79 hot spring metagenomes, which then were used for further analyses. Altogether these 21 cyanobacteria-containing metagenomes were distributed in North and South America, Asia and Antarctica (Figure 1), representing a temperature range between 32 and 75°C, and a pH range of 5.8 to 9.2. These comprised 359.8 Gbp of trimmed reads and 5.19 Gbp of assembled contigs (representing 1,393,425 contigs > 1,000 bp). A total of 1,152 medium- or high-quality MAGs were recovered from these 21 metagenomes. According to the GTDB-tk taxonomic assignment, most of these MAGs belong to the phyla Proteobacteria (16.1%), Chloroflexota (13.8%), Bacteroidota (12.9%), Planctomycetota (6.3%) and Cyanobacteria (4.9%) (Supplementary Table 5).

A total of 20 cyanobacterial MAGs (phylum Cyanobacteria GTDB R89) were recovered from the four new metagenomes reported in the present study. Overall, 57 cyanobacterial MAGs with an average completeness of 88.5% (SD ± 14.7), were obtained from the entire set of 21 hot spring metagenomes. The average contamination of these MAGs was 1.2% (SD ± 1.1), with 4.8% being the highest value (Table 1). According to the GSC quality parameters (Bowers et al., 2017), only two MAGs were classified as high-quality (M3746_SRR7905025_W2019_013 and M46_SRR2626160_R2017_013); while 55 MAGs were medium-quality, from which 36 could be categorized as high-quality if their rRNA operon sequences were binned.

**TABLE 1:** Genome features of hot spring cyanobacterial MAGs.

Genome ID	GTDB Classification	NCBI WGS Accession Number	Completeness (%)	Contamination (%)	Genome size (bp)	# Predicted CDS	Ribosomal RNAs
T60_TAT2020_004	c_Sericytochromatia;o_UBA7694;f_;g_;s_	JACYMC000000000	83.3	2.1	4167372	3865	nf
M55_SRR7473442_K2018_030	o_;f_;g_;s_	-	76.3	1.2	3104692	3433	nf
C42_CAH2020_026	o_Cyanobacteriales;f_Chroococcidiopsidaceae; g_Chroogloeocystis;s_Chroogloeocystis siderophila	-	87.8	0.7	4073818	4184	nf
T60_TAT2020_053	o_Cyanobacteriales;f_Cyanobacteriaceae; g_Cyanobacterium;s_	JACYMF000000000	99.1	0.0	3360103	3188	nf
M7585_ERR372908_C2015_104	o_Cyanobacteriales;f_Cyanobacteriaceae;g_Geminocystis;s_	DVEF00000000	98.7	0.2	2823523	2645	p
M4454_SRR7905024_W2019_049	o_Cyanobacteriales;f_Geitlerinemaceae;g_1;s_1	DVDY00000000	99.6	0.7	5103222	4498	nf
M59_SRR7905023_W2019_021	o_Cyanobacteriales;f_Geitlerinemaceae;g_1;s_1	DVED00000000	98.0	0.4	4985496	4422	p
M55_SRR7473442_K2018_032	o_Cyanobacteriales;f_Microcystaceae;g_;s_	-	60.0	1.1	2298071	3211	p
C42_CAH2020_068	o_Cyanobacteriales;f_Microcystaceae;g_Hydrococcus; s_Hydrococcus minor	JACYLS000000000	99.3	0.2	4917303	4538	nf
C42_CAH2020_038	o_Cyanobacteriales;f_Nostocaceae;g_Calothrix;s_	JACYLQ000000000	99.8	0.5	6532080	5465	nf
C42_CAH2020_084	o_Cyanobacteriales;f_Nostocaceae;g_Chlorogloeopsis; s_Chlorogloeopsis fritschii	JACYLT000000000	99.3	1.1	7052188	6270	nf
M7585_ERR372908_C2015_036	o_Cyanobacteriales;f_Nostocaceae;g_Fischerella;s_	-	80.2	2.4	4599829	4836	nf
C42_CAH2020_099	o_Cyanobacteriales;f_Nostocaceae;g_Fischerella;s_Fischerella thermalis	-	51.4	3.7	3171434	3795	nf
M33_SRR5581334_DOE_064	o_Cyanobacteriales;f_Nostocaceae;g_Fischerella;s_Fischerella thermalis	-	76.7	1.2	4199934	4157	p
M48_SRR5451033_A2018_028	o_Cyanobacteriales;f_Nostocaceae;g_Fischerella;s_Fischerella thermalis	JACYLX000000000	98.6	0.2	5141023	4455	nf
M58_SRR5451032_A2018_009	o_Cyanobacteriales;f_Nostocaceae;g_Fischerella;s_Fischerella thermalis	JACYLY000000000	97.5	0.2	5203860	4497	nf
M66_SRR5451031_A2018_004	o_Cyanobacteriales;f_Nostocaceae;g_Fischerella;s_Fischerella thermalis	JACYMA000000000	98.8	0.0	5271540	4589	nf
T60_TAT2020_040	o_Cyanobacteriales;f_Nostocaceae;g_Rivularia;s_	JACYMD000000000	99.1	0.7	6018129	5143	p
M33_SRR5581334_DOE_039	o_Cyanobacteriales;f_Nostocaceae;g_Trichormus;s_	DVDT00000000	99.3	0.4	7018248	5764	nf
M33_SRR5581334_DOE_052	o_Cyanobacteriales;f_Oscillatoriaceae;g_1;s_1	DVDU00000000	99.3	0.8	6239312	5233	p
M59_SRR7905023_W2019_015	o_Cyanobacteriales;f_Oscillatoriaceae;g_2;s_1	-	98.6	1.4	5787682	4574	nf
M7585_ERR372908_C2015_266	o_Cyanobacteriales;f_Oscillatoriaceae;g_3;s_1	DVEG00000000	99.6	0.0	3979146	3395	p
K32_KRO2020_035	o_Elainellales;f_1;g_1;s_1	JACYLV000000000	98.6	1.1	4371702	3742	nf
K44_KRO2020_017	o_Elainellales;f_1;g_1;s_1	JACYLW000000000	99.1	1.1	4432392	3767	nf
M4564_SRR6941191_B2018_003	o_Elainellales;f_1;g_1;s_2	-	54.1	2.3	2340930	3827	p
T60_TAT2020_003	o_Elainellales;f_1;g_1;s_3	JACYMB000000000	95.2	0.8	4020528	3982	nf
C42_CAH2020_086	o_Elainellales;f_Elainellaceae;g_1;s_1	JACYLU000000000	99.5	1.2	5928601	5059	nf
M58_SRR5451032_A2018_015	o_Elainellales;f_Elainellaceae;g_1;s_1	JACYLZ000000000	98.9	1.9	5813440	5026	nf
M66_SRR5451031_A2018_013	o_Elainellales;f_Elainellaceae;g_1;s_1	-	66.7	1.4	3168891	3064	nf
M55_SRR7473442_K2018_004	o_Elainellales;f_Elainellaceae;g_2;s_1	DVEB00000000	97.9	1.4	5013995	4164	nf
M4564_SRR6941191_B2018_001	o_Elainellales;f_Elainellaceae;g_CCP2;s_	-	87.6	2.7	6826097	8104	c
C42_CAH2020_014	o_Elainellales;f_Elainellaceae;g_Elainella;s_1	-	92.7	0.9	6368923	5661	nf
C42_CAH2020_052	o_Elainellales;f_Elainellaceae;g_Elainella;s_2	-	82.6	1.3	6281096	5592	nf
M66_SRR5451031_A2018_017	o_Elainellales;f_Elainellaceae;g_Elainella;s_2	-	76.4	4.8	6099463	5906	nf
C42_CAH2020_010	o_Elainellales;f_Elainellaceae;g_Elainella;s_Elainella sp000733415	JACYLO000000000	99.5	1.0	7459088	6354	p
M55_SRR7473442_K2018_002	o_Elainellales;f_Elainellaceae;g_O-77;s_	DVEA00000000	97.6	1.2	5022360	4498	nf
C42_CAH2020_037	o_Elainellales;f_Elainellaceae;g_O-77;s_O-77 sp001548395	JACYLP000000000	99.1	1.2	5291374	4455	nf
M33_SRR5581334_DOE_111	o_Gloeobacterales;f_Gloeobacteraceae;g_;s_	-	57.4	0.0	3328352	4052	nf
C42_CAH2020_066	o_Gloeomargaritales;f_Gloeomargaritaceae;g_;s_	JACYLR000000000	99.1	2.6	2730382	2813	nf
M33_SRR5581334_DOE_097	o_Leptolyngbyales;f_Leptolyngbyaceae;g_;s_	DVDV00000000	99.5	0.7	6960501	6277	nf
T60_TAT2020_044	o_Leptolyngbyales;f_Leptolyngbyaceae;g_Alkalinema;s_	-	86.3	2.0	3677935	4081	nf
C42_CAH2020_001	o_Leptolyngbyales;f_Leptolyngbyaceae;g_JSC-12;s_1	JACYLN000000000	99.5	0.0	5550306	5066	nf
M33_SRR5581334_DOE_055	o_Leptolyngbyales;f_Leptolyngbyaceae;g_JSC-12;s_2	-	68.1	4.8	4528759	5838	nf
M65_SRR7473443_K2018_014	o_Leptolyngbyales;f_Leptolyngbyaceae;g_JSC-12;s_3	-	62.3	2.2	3370404	4476	nf
M44_SRR5580903_DOE_062	o_PCC-7336;f_JA-3-3Ab;g_JA-3-3Ab;s_1	DVDX00000000	100.0	0.0	2925579	2737	nf
M46_SRR5216251_DOE_021	o_PCC-7336;f_JA-3-3Ab;g_JA-3-3Ab;s_2	-	71.2	1.9	1764837	2077	nf
M60_SRR5451356_T2016_033	o_PCC-7336;f_JA-3-3Ab;g_JA-3-3Ab;s_3	-	56.4	1.4	1200192	1708	nf
M60_SRR5248366_T2016_029	o_PCC-7336;f_JA-3-3Ab;g_JA-3-3Ab;s_JA-3-3Ab sp000013205	-	77.6	0.9	1957034	2127	nf
M60_SRR5451356_T2016_037	o_PCC-7336;f_JA-3-3Ab;g_JA-3-3Ab;s_JA-3-3Ab sp000013205	-	92.1	0.9	2379147	2435	nf
M60_SRR5248366_T2016_047	o_PCC-7336;f_JA-3-3Ab;g_JA-3-3Ab;s_JA-3-3Ab sp000013225	-	91.8	0.4	2358112	2481	nf
M60_SRR5451356_T2016_004	o_PCC-7336;f_JA-3-3Ab;g_JA-3-3Ab;s_JA-3-3Ab sp000013225	-	88.6	1.2	2201305	2331	nf
M65_SRR7473443_K2018_010	o_Phormidesmiales;f_Phormidesmiaceae;g_1;s_1	DVEE00000000	95.4	3.0	3894922	3941	nf
T60_TAT2020_046	o_Phormidesmiales;f_Phormidesmiaceae;g_2;s_1	JACYME000000000	97.8	0.5	4202921	3943	nf
M46_SRR2625865_R2017_001	o_Thermosynechococcales;f_Thermosynechococcaceae; g_Thermosynechococcus;s_1	-	63.1	1.4	1882589	3438	p
M46_SRR2626160_R2017_013	o_Thermosynechococcales;f_Thermosynechococcaceae; g_Thermosynechococcus;s_1	DVDZ00000000	96.9	1.1	2396365	2707	c
M55_SRR7473442_K2018_012	o_Thermosynechococcales;f_Thermosynechococcaceae; g_Thermosynechococcus;s_2	DVEC00000000	97.8	0.2	2399209	2384	p
M3746_SRR7905025_W2019_013	o_Thermosynechococcales;f_Thermosynechococcaceae; g_Thermosynechococcus;s_Thermosynechococcus sp000505665	DVDW00000000	98.1	0.0	2387168	2307	c

*Features were determined with the CheckM tool (completeness, contamination, genome size and number of CDSs), barrnap (ribosomal RNAs: nf, not found; p, partial and c, complete 5S-16S-23S subunits) and GTDB-tk R89 (GTDB classification). MAGs are ordered according to the GTDB-tk classification and their NCBI WGS Accession numbers are given for the 34 MAGs submitted to the NCBI.*

Several strategies have been used to recover partial genomes of hot spring cyanobacteria from metagenomes, including mapping contigs against reference genomes (Bhaya et al., 2007) and the use of binning tools (Thiel et al., 2017; Alcorta et al., 2018; Ward et al., 2019). Metagenomes from the binning-based studies were reanalyzed here, allowing for better genome recovery in terms of contig numbers and completeness. Furthermore, the ssu_erroneous module from refineM enabled removal of partial 16S rRNA gene sequences with incongruent taxonomy from other phyla. Reanalysis of previously published data is useful for maintaining confidence in public repositories of genomes (Shaiber and Eren, 2019) and to obtain genomes that fulfill acceptable standards (Bowers et al., 2017).

### Hot Spring Cyanobacterial Taxonomy

Cyanobacterial taxonomy and nomenclature have always been controversial (see Oren and Ventura, 2017, and references therein). Nowadays, applying a valid taxonomy based on the genomes of uncultured organisms is impossible (e.g., the rejection of the Whitman (2016) proposal), even when following good practice strategies (Konstantinidis et al., 2017; Chun et al., 2018). This study increased the percentage of available genomes within cyanobacterial taxa that have few cultured representatives that hopefully can be cultivated in the future.

**FIGURE 2:**
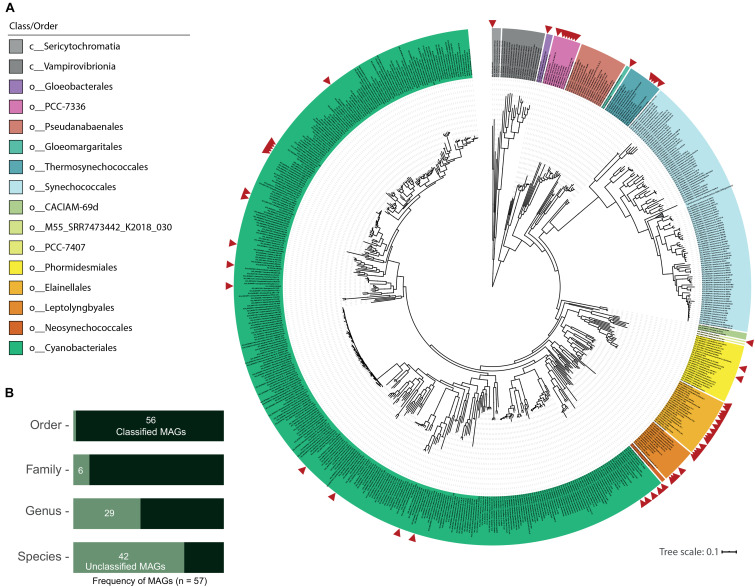
Novel hot spring cyanobacterial MAG phylogeny and taxonomic classification. **(A)** Phylogenomic reconstruction from multiple sequence alignment of 120 bacterial marker genes recovered from 800 NCBI cyanobacterial genomes and 57 new cyanobacterial MAGs from 21 hot spring metagenomes. Maximum likelihood tree reconstruction was performed with IQtree software using the LG + R10 model and a non-parametric UF-bootstrap support of 1,000 replicates. The labels are colored according to the GTDB order classification for c_Cyanobacteriia genomes, and class for Sericytochromatia and Vampirovibrionia members. The tree was rooted in the node between Sericytochromatia/Vambirovibrionia and Cyanobacteriia classes. 305 sequences in the Syn/Pro clade are collapsed in the tree. Red arrows indicate the position of hot spring MAGs in the tree that were distributed into 10 orders from a total of 15 in the cyanobacterial phylum. The new order retrieved in the present study is called M55_SRR7473442_K2018_030 in the legend. **(B)** Classification of MAGs according to the GTDB-tk. Unclassified MAGs correspond to those with no associated genome in the database at the specific taxonomic level.

As seen in Figure 2A, GTDB classification distributed the new 57 MAGs into 2 classes and 10 orders within the phylum Cyanobacteria (from 13 total orders in the class Cyanobacteriia and 2 orders in the class Sericytochromatia in GTDB R89). This dataset includes one MAG (medium-quality) classified as a new order in the Cyanobacteriia class (M55_SRR7473442_K2018_030: 76.3% completeness and 1.2% contamination). Only T60_TAT2020_004 was assigned to the non-photosynthetic Sericytochromatia class. ANI values between all MAGs distinguished 44 different species (Table 1), while GTDB-tk classified 14 MAGs to species with available genomes in the NCBI database (gANI > 95%; 9 different species when the MAGs were dereplicated; Table 1 and Figure 2B). Additionally, 21 MAGs were classified by topology or RED values as new species belonging to established GTDB genera. In summary, 43 MAGs represent taxonomic novelty as 1 new order, 3 new families, 15 new genera and 36 new species (Table 1).

This MAG classification is supported by a maximum likelihood phylogenomic reconstruction that incorporated 120 bacterial marker genes (Figure 2A) and included 800 NCBI cyanobacterial genomes (> 75% completeness; dereplicated from 1,626 genomes available in the NCBI assembly database as of September 2019). Distribution of the 57 MAGs in the GTDB R89 orders is as follows: o__ (new, 1), Cyanobacteriales (20), Elainellales (15), Gloeobacterales (1), Gloeomargaritales (1), Leptolyngbyales (5), PCC-7336 (7), Phormidesmiales (2), Thermosynechococcales (4) and UBA7694 (1, from the Sericytochromatia class). Some of these genomes form clades with known thermophilic cyanobacterial species from the following genera: *Fischerella*, *Thermosynechococcus*, *Synechococcus* (JA-3-3Ab), *Chroogloeocystis*, *Chlorogloeopsis*, *Calothrix*, *Hydrococcus* and O-77 (*Thermoleptolyngbya*). Others represent the first hot spring-associated genome reported within the given genus, specifically *Rivularia* (3 marine genomes), *Alkalinema* (1 freshwater genome), CCP2 (1 saltern genome) and *Cyanobacterium* (3 freshwater/saline lake genomes), as seen in Supplementary Table 3. The latter supports the concept that the ability to live at higher temperatures is a secondary specialization from primordial environments like marine or fresh water (Hammerschmidt et al., 2020), even in early-branching cyanobacteria, such as the Thermosynechococcales and PCC7336 orders, that now include non-thermal cyanobacterial members as revealed by GTDB classification (Supplementary Figure 1).

Cyanobacterial clades with few genomes are complemented by the group of MAGs that comprises new taxa at the genus or family levels, according to GTDB (see above). For instance, some of these new genomes are related to the recently described Gloeomargaritales order, whose members can accumulate intracellular carbonates (Moreira et al., 2017; Ponce-Toledo et al., 2017), as well as the early diverging non-thylakoid Gloeobacterales (Nakamura et al., 2003). Furthermore, the MAG from the non-photosynthetic Sericytochromatia class (UBA7694 order) further expands the described habitat of this taxon (Monchamp et al., 2019). This MAG also shows other features that are not currently associated with UBA7694 family genomes, such as the potential for H_2_ metabolism and a higher number of CRISPR-associated proteins (see below). Altogether, this data provides new insight into the diversity of thermophilic, thermotolerant (< 40°C) and hot spring-associated cyanobacteria, and hints at the yet-to-be-discovered taxonomic novelty in these extreme environments (e.g., the large numbers of unassigned cyanobacterial sequences from hot springs reported in Roeselers et al., 2007; Ionescu et al., 2010; Mackenzie et al., 2013; Momper et al., 2019; Uribe-Lorío et al., 2019, among others).

Although GTDB taxonomy solves several of the problems and rearrangements within the phylum, there are still unsolved issues. One of them is the use of placeholder names, such as the O-77 genus that should correspond to *Thermoleptolyngbya* (Sciuto and Moro, 2016; Yoon et al., 2017); the JSC-12 genus that has no generic assignment; and the JA-3-3Ab genus that is mostly known as *Synechococcus* OS-A and OS-B’ (Steunou et al., 2006; Bhaya et al., 2007). Moreover, the present phylogenomic analyses using GTDB R89 reproduced the polyphyly within the genera *Chroogloeocystis* (Brown et al., 2005), *Geminocystis* (Korelusova et al., 2009), *Calothrix* (Berrendero et al., 2008) and *Synechococcus* (Walter et al., 2017). Further actions should now focus on selecting and correctly updating the type species and type material to improve cyanobacterial taxonomy (Ramos et al., 2017). This will enable more taxonomically driven sequencing efforts like cyanoGEBA (Shih et al., 2013), as well as the inclusion of genomic type material from uncultured taxa (Chuvochina et al., 2019), such as the MAGs recovered in this study.

### Cyanobacterial MAG Abundances in Hot Spring Metagenomes

**FIGURE 3:**
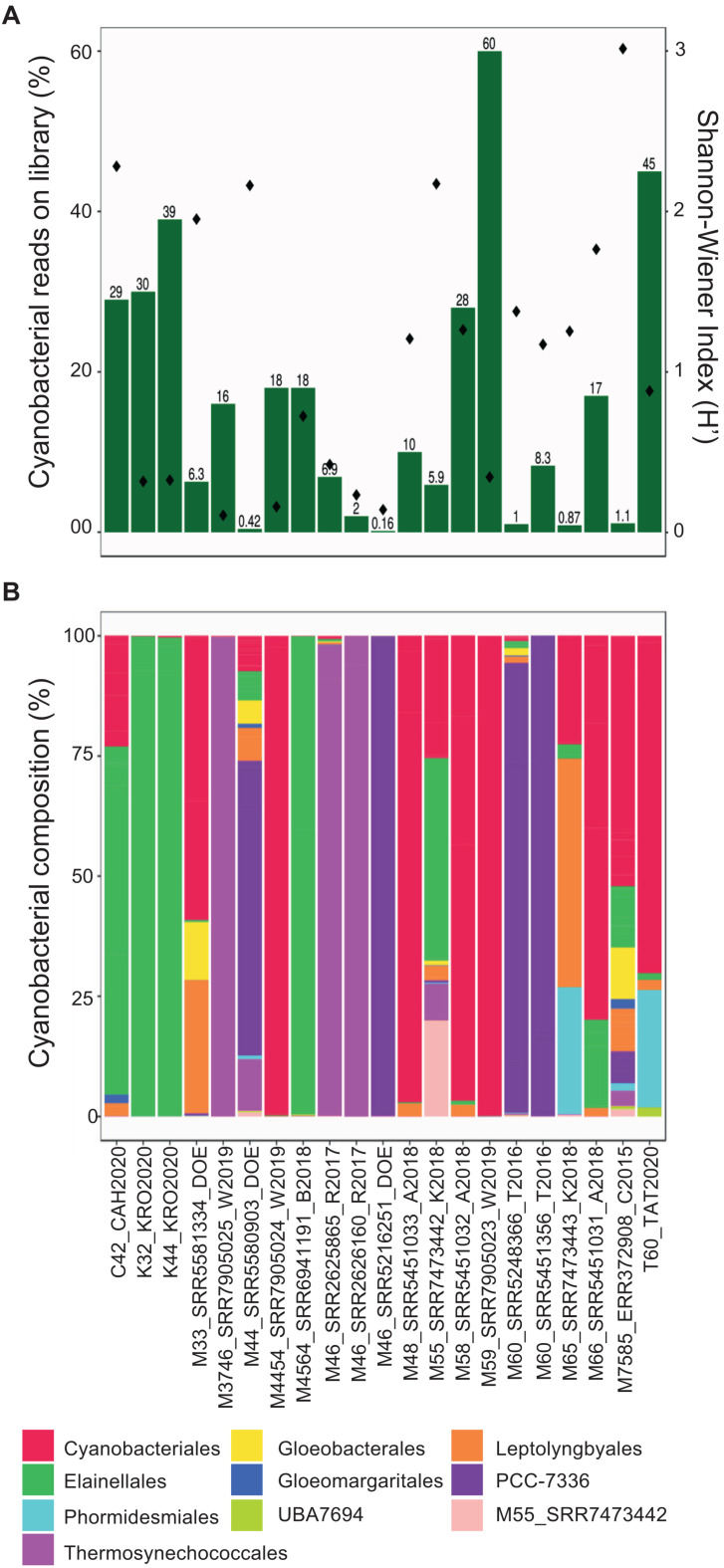
Diversity of hot spring cyanobacterial MAGs in metagenomes. **(A)** Left *y*-axis corresponds to the percentage of total mapped reads from each hot spring to a cyanobacterial MAG (green bars). Right *y*-axis corresponds to alpha-diversity values (dots) for each metagenome. **(B)** Normalized (relative) abundances of the reads assigned to each MAG at the order level in each sample. The possible new order is shown as M55_SRR7473442.

Taxonomic annotation is relevant for all cyanobacterial MAGs recovered here, but especially for those that may have a global or dominant distribution in hot springs. Accordingly, the proportion of MAG reads mapped against the entire set of metagenomes was determined and ranged from 0.1 to 60% (Figure 3A). The most represented orders (35 to 99% of the total cyanobacterial community) were Cyanobacteriales, Elainellales, Thermosynechococcales and PCC-7336 (Figure 3B and Supplementary Figure 3). The abundance of these MAGs in each hot spring was negatively correlated with the overall cyanobacterial alpha diversity in the respective metagenome (−0.490 rho Spearman’s correlation, *p*-value < 0.05; Figure 3A and Supplementary Figure 2A). The latter suggests that in low diversity systems, where cyanobacteria represent a major proportion of the microbial community, only a few MAGs outcompete other cyanobacterial species and successfully establish themselves as the dominant producers in the system. Whereas, in samples where non-cyanobacterial taxa dominate the community, a broader range of niches might be available for different groups of cyanobacteria to colonize.

Interestingly, multivariate analyses, which were used to investigate the relationship between abundances, physico-chemical and geographic features, revealed that the cyanobacterial community composition was positively correlated with the hot spring location (Mantel statistic = 0.19, *p*-value = 9 × 10^–4^), but not with physicochemical parameters, such as temperature or pH (Mantel test, *p*-value > 0.01). For example, MAGs from the PCC-7336 order were more abundant in North American samples, while Thermosynechococcales MAGs were found mainly in Asian samples independent of hot spring conditions (Supplementary Figure 3), thereby corroborating the proposed biogeographical islands for these unicellular cyanobacteria (Papke et al., 2003; Cheng et al., 2020). The correlation between community composition and geographical location was also observed through non-metric multidimensional scaling and cluster analyses (Supplementary Figures 2B,C). Furthermore, the cyanobacterial community composition presented a similar pattern even within a specific hot spring, independent of the temperature and pH (Supplementary Figure 2C).

Another interesting result is that a single clade commonly dominates the cyanobacterial community within each hot spring, where a single order can make up to 99% of all cyanobacterial sequences (Figure 3B). Altogether, this information notices the possibility that different hot spring cyanobacteria occupy a similar niche in non-acidic thermal environments and that competitive exclusion between different cyanobacterial clades is a significant force driving the cyanobacterial community composition, as seen in saline freshwater systems (Roney et al., 2009). Accordingly, some hot springs have zones with different dominant cyanobacteria, sugesting successful competitive exclusion depending on the nitrogen compound availability, sulfide concentration, temperature resistance of each clade, and light adaptations in the thermal gradient (Ward et al., 2012). However, more studies are needed to test the competitive exclusion in these different cyanobacterial communities.

### Comparative Genomics: General Features of Genomes and Proteins

Large-scale comparisons have shown that there are common distinctive genomic features that differentiate thermophilic archaea and bacteria from their mesophilic counterparts; however, thermophilic cyanobacteria were underrepresented in these analyses (Singer and Hickey, 2003; Sabath et al., 2013). The cyanobacterial comparative genomics carried out in the present study, both at a general level (the 159-subset) and at specific taxonomic levels (families and genera), was useful to confirm previously detected genome trends of some cyanobacteria, such as genomic streamlining toward extreme environments (Larsson et al., 2011).

**FIGURE 4:**
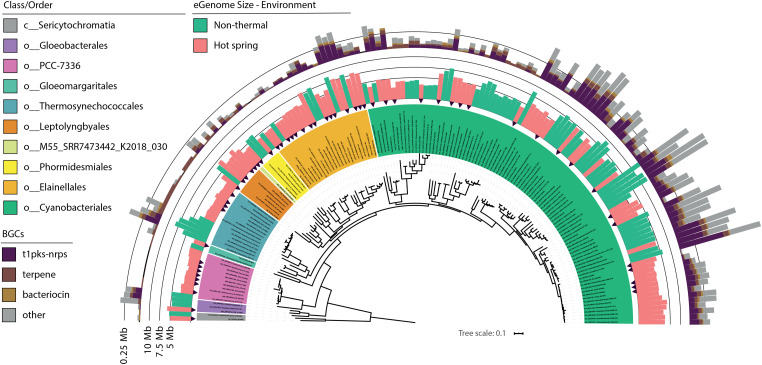
Genome size and secondary metabolite clusters of hot spring and close non-thermal cyanobacteria. Phylogenomic reconstruction from multiple sequence alignment of 120 bacterial marker genes recovered from 102 NCBI cyanobacterial genomes and 57 cyanobacterial MAGs from 21 hot spring metagenomes. Maximum likelihood tree reconstruction was done with IQtree software using the LG + R10 model and a non-parametric UF-bootstrap support of 1,000 replicates. The labels are colored according to the GTDB order classification for c_Cyanobacteriia genomes and class for Sericytochromatia members. The tree was rooted in the node between the Sericytochromatia and Cyanobacteriia classes. Black arrows indicate the position of hot spring MAGs in the tree. The inner ring indicates the estimated genome size (Mbp) for all 159 genomes and is colored according to the respective environmental origin (see Supplementary Table 3). The outer ring depicts the total size (Mbp) of secondary metabolite biosynthetic gene clusters (BGCs) detected for each genome using antiSMASH software.

**FIGURE 5:**
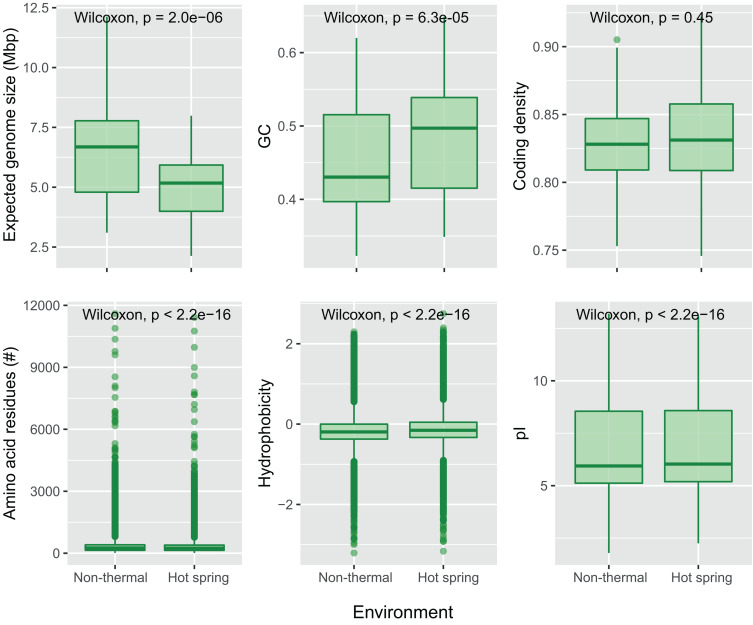
Comparison of genomic and protein properties between hot spring and non-thermal genomes. Boxplots for the “expected genomes size,” GC content and coding density were compared for the 159-subset genomes (93 from hot springs and 66 from non-thermal environments). The number of amino acid residues, hydrophobicity and isoelectric point (pI) were compared for the predicted CDSs from the 159-subset genomes from which 369,249 were from hot spring genomes and 348,018 from non-thermal genomes. Wilcoxon’s paired test was performed for each dataset, and *p*-values with Bonferroni’s multiple test correction are reported.

For the present study, the trend of an increasing optimum growth temperature with a decreasing genome size (Sabath et al., 2013) was also confirmed as a negative correlation between the source temperature of the metagenome and the expected genome size of the cyanobacterial MAGs (−0.355 Pearson’s correlation, *p*-value < 0.05). Differences in genome size and other features were compared between hot spring and non-thermal cyanobacterial genomes of the 159-subset, which comprised the 57 MAGs, 36 NCBI hot spring genomes and 66 NCBI non-thermal genomes, see section “MATERIALS AND METHODS” and Supplementary Table 3. Cyanobacterial MAGs showed a wide variation in genome size from 2.1 to 12.1 Mbp across the 10 different orders (Figure 4). However, hot spring cyanobacterial genomes (*n* = 93) were smaller and exhibited a higher GC percentage (Wilcoxon’s paired test, *p*-value < 0.05 for both analyses) than the non-thermal genomes (*n* = 66), while the coding density was similar between both groups (Wilcoxon’s paired test, *p*-value = 0.15) (Figure 5).

Additionally, whether or not some of these genome changes were reflected in the divergence of cyanobacterial genera and families with ≥ 3 genomes in each environmental group was investigated. For the genus *Fischerella*, hot spring genomes (*n* = 16) were 1.6 (± 0.7) Mbp smaller than the non-thermal genomes (*n* = 6) and had a higher GC content (Supplementary Figures 4, 5, Wilcoxon’s paired test, *p*-value < 0.05). Similarly, the families *Elainellaceae* and *Thermosynechococcaceae* exhibited smaller hot spring genomes (Wilcoxon’s paired test, *p*-value < 0.05; Supplementary Figure 4), with an increase in both the GC content and coding density for *Thermosynechococcaceae* (Wilcoxon’s paired test, *p*-value < 0.05; Supplementary Figures 5, 6). However, the low number of genomes for other genera and families in either environmental group allowed for only some trends to be observed. *Alkalinema* and *Rivularia* genomes from hot springs were > 2 Mbp smaller than those from non-thermal environments, and a similar trend was observed for the JA-3-3Ab order. Specifically, genomes of the JA-3-3Ab family (*Synechococcus* sp. OS-A and OS-B’ clade) were 2.4 (± 0.3) Mbp smaller than the marine *Synechococcus* sp. PCC 7336 genome from the same order.

Furthermore, differences in nucleotide content and protein properties that have been seen in other bacteria (Singer and Hickey, 2003; Sabath et al., 2013) were also found here. Analysis of the coding DNA sequences (Figure 5, Wilcoxon’s paired test, *p*-values < 0.05) showed that hot spring cyanobacterial proteins (*n* = 369,249) are not only shorter than those from non-thermal environments, but also lighter in molecular weight, more hydrophilic, and contain more basic isoelectric points. Indeed, amino acid composition analysis showed that hot spring cyanobacterial proteins have more basic amino acids, such as histidine and arginine, and less acidic amino acids, such as aspartate and glutamate. The aromatic tryptophan and the non-polar residues alanine, leucine, proline and valine were also more abundant in hot spring genomes. The increased alanine abundance (0.81%) and decreased asparagine (0.63%) and lysine (0.62%) abundances were the most notable differences (Wilcoxon’s paired test *p*-values < 0.05, Supplementary Figure 7), explaining the tendency toward more hydrophobic proteins in hot spring genomes. These amino acid frequencies agree with previous predictions of increased arginine and valine, and decreased serine and aspartate abundances in other thermophiles and hyperthermophiles (Vieille and Zeikus, 2001; Singer and Hickey, 2003), but disagree for the other amino acid frequencies observed here.

### Comparative Genomics: Orthologous Sequences

Analysis of orthologous CDSs was conducted in the 159-subset to determine if there were genes exclusively related to hot spring genomes. Of the 719,564 analyzed protein sequences, 691,501 (96.1%) were assigned into 47,328 orthogroups, 59.2% of which were singletons. Only 183 (0.4%) orthogroups had at least one ortholog sequence in ≥ 97% of the analyzed genomes, while 12,657 (26.7%) were sporadically present in both environments. The number of orthogroups with only orthologs (> 1, not singletons) in non-thermal genomes was 3,434 (7.3%, 66 genomes), while that from hot springs was 3,179 (6.7%, 93 genomes). Since environment-specific orthogroups with CDSs across taxa point to potential niche adaptation, CDSs present in more than one class, family or genus were searched. Several orthogroups were shared within 6 different orders or 7 families in hot spring genomes, while non-thermal genomes shared fewer orthogroups at these taxonomic levels (Supplementary Figure 8). Most of these widely distributed orthogroups from hot spring genomes were annotated as hypothetical proteins, but other annotated functions included a DsrE family protein and an SDR family oxidoreductase (Supplementary Table 6). Phylogenetic analysis of hypothetical protein orthogroups OG0008066 (shared between 5 families) and OG0006223 (shared between 7 genera) not only corroborates the close relationship between hot spring cyanobacteria, but also demonstrates an association with other common thermophilic bacteria, such as Chloroflexota, Deinococcota and Actinobacteriota (Supplementary Figures 9A,B). The distribution of these genes across diverse hot spring bacteria could be explained by horizontal gene transfer, as also found between other organisms living at high temperatures (Fuchsman et al., 2017).

**FIGURE 6:**
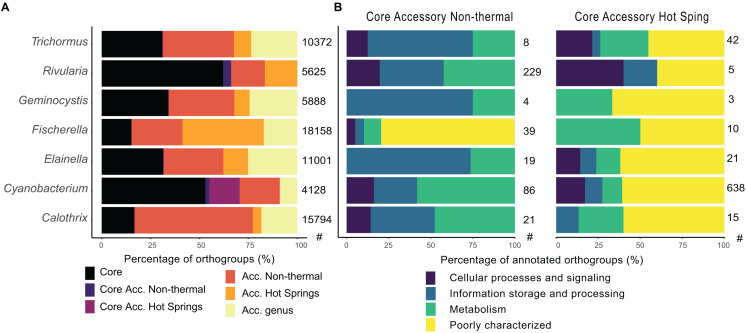
Comparison of core and accessory orthogroups from hot spring and non-thermal cyanobacterial genera. **(A)** The classification of orthogroup fractions according to their presence in all (core), in only hot spring or non-thermal genomes (specific accessory), or in both groups at the genus level (accessory genus). **(B)** COG characterization by eggnog mapper software of exclusive core accessory genes of hot spring and non-thermal environments for each compared genus. The categories were joined in the four main COG functional groups. The number of hot spring (HS) and non-thermal (NT) genomes used for these analyses is as follows: *Trichormus* (2 HS, 6 NT), *Rivularia* (1 HS, 3 NT), *Geminocystis* (3 HS, 3 NT), *Fischerella* (16 HS, 6 NT), *Elainella* (5 HS, 2 NT), *Cyanobacterium* (1 HS, 3NT) and *Calothrix* (2 HS, 8 NT). The number of orthogroups for each analysis is shown in the right side of each bar plot.

Changes within the hot spring core and accessory genomes were determined for 7 genera (*Calothrix*, *Cyanobacterium*, *Elainella*, *Fischerella*, *Geminocystis*, *Rivularia*, and *Trichormus*) with both hot spring and non-thermal representatives. The core genome of each genus ranged from 2,007 to 3,488 CDSs, representing 24% (*Calothrix*) to 75% (*Geminocystis* and *Cyanobacterium*) of the CDSs in a genome. The orthogroups were classified according to whether they were present in all genomes (core), exclusively in hot spring or non-thermal genomes (specific accessory and specifc core accessory sets), or genus accessory if they were sporadically present in both environmental groups (Figure 6A and Supplementary Table 7). The COG distribution (Supplementary Figure 10) shows core genomes clustering, along with some of the accessory orthogroups from hot spring and non-thermal genomes. Core accessory orthogroups for all hot spring and non-thermal genera (except for *Cyanobacterium*) clustered together, suggesting common functions associated with these groups.

Regarding gene annotation (Figure 6B), non-thermal core accessory orthogroups had more COG function abundances in all genera that were classified as unknown (31, Poorly Char.); replication and repair (24, Information Stor.); energy production and metabolism (22, Metabolism); cell wall/membrane biogenesis (17, Cell Proc.); transcription (17, Information Stor.); and coenzyme metabolism (14, Metabolism) (except for the genus *Rivularia*, whose most represented function was transcription with 54 core accessory orthogroups in the non-thermal genomes). Nevertheless, core accessory genes of hot spring genomes did not exceed 42 orthogroups for each genus, with functions mostly classified in all genera as unknown (50, Poorly Char.); inorganic ion transport (8, Metabolism); amino acid metabolism and transport (6, Metabolism); and cell wall/membrane biogenesis (5, Cell Proc.). For the genus *Cyanobacterium*, which had only one available hot spring genome, most represented COGs in the hot spring core accessory orthogroups were related to unknown functions (388) (Figure 6B).

In general, similar patterns were found for the functional distribution of core genomes between cyanobacterial genera in both the hot spring and non-thermal environments, thereby corroborating previous analyses (Beck et al., 2012). However, these results also show a higher proportion of genes with unknown function in the core accessory genomes of hot spring cyanobacteria. The low number of identified core accessory genes might suggest a reductionist point of view for adaptation to high temperatures, as previously seen for the thermophilic unicellular red algae *Galdieria sulphuraria*, whose adaptation to this environment was mediated by horizontal gene transfer from bacteria and archaea (Schönknecht et al., 2013).

### Comparative Genomics: Differences in Functional Categories

**FIGURE 7:**
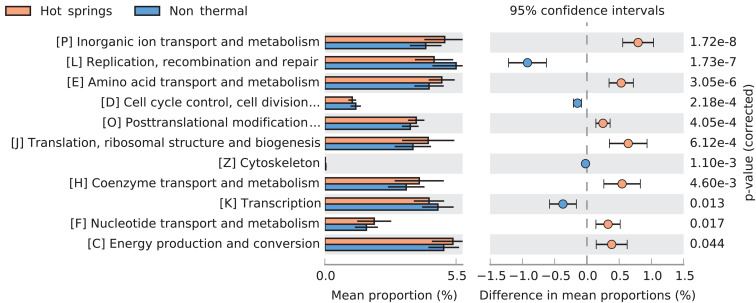
Comparison of COG categories between hot spring and non-thermal genomes. Extended error bar plot showing significantly different predicted COG categories between hot spring (*n* = 93) and non-thermal (*n* = 66) cyanobacterial genomes. Only corrected p-values < 0.05 are displayed.

Several differences in COG categories were found between the 159-subset of hot spring and non-thermal genomes (Wilcoxon’s paired test, *p*-value < 0.05). The greatest difference was a decrease (∼1% relative abundance) in hot spring genomes for the L COG category (replication, recombination and repair). Additionally, higher abundances were detected in categories P (inorganic ion transport and metabolism), J (translation, ribosomal structure and biogenesis), H (coenzyme transport and metabolism), E (amino acid transport and metabolism) and O (post-translational modification, protein turnover and chaperones). Conversely, other COG categories, such as K (transcription), D (cell cycle control, cell division and chromosome partitioning) and Z (cytoskeleton) decreased in hot spring genomes (Figure 7).

An additional comparison of specific functions related to complementary metabolisms and genes involved in microbial community interactions was performed. The results show no differences for the sulfide-quinone reductase gene (related to anoxygenic photosynthesis; Chi-square test *p*-value = 0.930) or for the *nif*HDK complex and accessory genes (related to nitrogen fixation; Wilcoxon’s paired test, *p*-value = 0.061). However, the potential acquisition of nitrate and its subsequent reduction to ammonium through the *nar*B and *nir*A genes were less represented in hot spring genomes (Wilcoxon’s paired test, *p*-value < 0.05), as were the *hox*, *hup*, *hyp*, and *hyb* genes related to hydrogen metabolism (Wilcoxon’s paired test, *p*-value < 0.05), supporting the competitive exclusion trend by specific nutritional adaptations in the thermal gradient (Ward et al., 2012).

**FIGURE 8:**
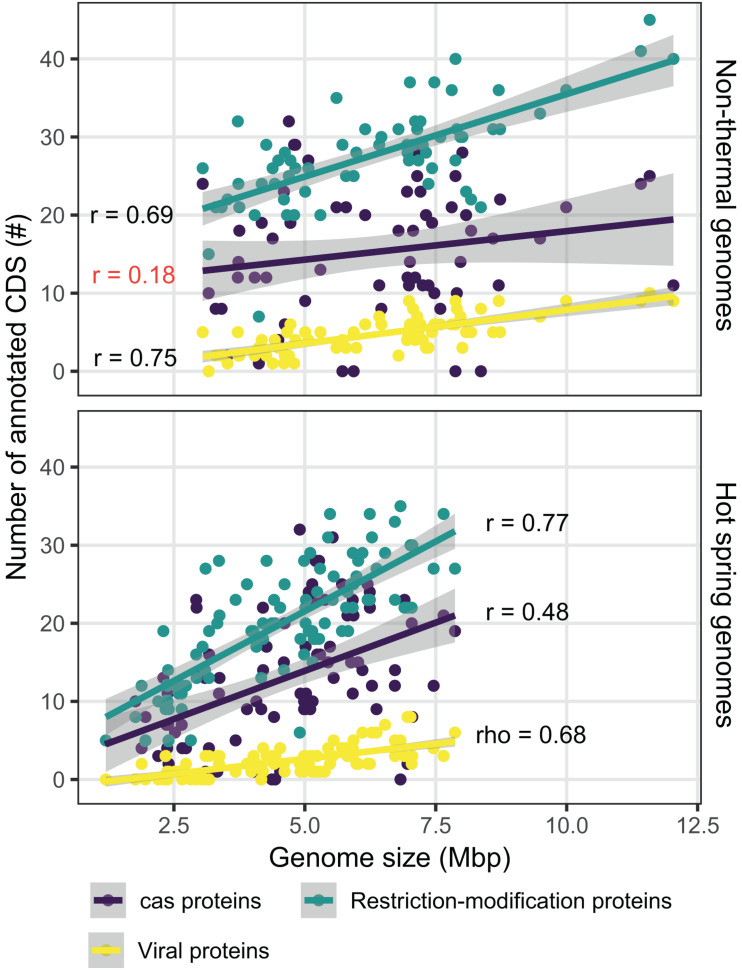
Comparison of defense mechanism-associated genes from hot spring and non-thermal genomes. Relationship between the number of CDSs determined for Cas proteins (purple), viral proteins (yellow) and restriction modification proteins (light blue), and the size of the 159 genomes used for hot spring/non-thermal comparisons. Upper panel shows non-thermal genomes (*n* = 66), while the bottom panel shows hot spring genomes (*n* = 93). Smooth linear regression was determined using R software with the ggplot package. Pearson’s r and Spearman’s rho correlations are provided for each protein category in the plot. Red represents correlations with a *p*-value > 0.05 and black represents those with *p*-values < 0.05.

Genes related to the defense mechanisms against foreign nucleic acids and integrated viral proteins were also compared. The number of annotated viral protein orthogroups was 18, showing broad integration of phage CDSs, except for 15 of the hot spring genomes (including all JA-3-3Ab members). Restriction-modification CDSs were classified into 131 different orthogroups, while CRISPR-associated proteins (Cas) were classified into 73 clusters. Considering that genome streamlining was a primary difference between the environmental groups, the correlation between genome size and the number of CDSs annotated with these functions was analyzed. The correlation was higher for restriction-modification and viral proteins (0.778 Pearson’s correlation, *p*-value < 0.05; and 0.747 rho Spearman’s correlation, *p*-value < 0.05, respectively; Figure 8), when compared to the Cas proteins (0.360 Pearson’s correlation, *p*-value < 0.05). This scenario varies for Cas proteins when the genomes are separated into hot spring and non-thermal environments (Figure 8), in that the Pearson’s correlation for hot spring genomes was 0.477 (*p*-value < 0.05), while the non-thermal genomes showed a lower and non-significant Pearson’s correlation index of 0.182 (*p*-value = 0.142). Conversely, restriction-modification and viral proteins presented similar Pearson’s correlation indexes between both environmental groups (Fischer r-to-z transformation *p*-values > 0.05).

Additionally, BGCs were analyzed because they have been found to be gained and lost during niche transitions (Kurmayer et al., 2015). A total of 6,773,751 bp representing secondary metabolite biosynthetic regions were identified for the 57 MAGs (varying from 0.08 to 11.15% of the total genome). The NRPS-PKS, bacteriocin and terpenes clusters were the most abundant (Figure 4), as has been previously seen for some hot spring cyanobacteria (Micallef et al., 2015). BGCs represented a smaller percentage in hot spring genomes (3.07 ± 2.6%, Wilcoxon’s paired test, *p*-value < 0.05) than in non-thermal genomes (5.18 ± 3.39%). For instance, *Fischerella* members show the greatest difference (0.5 ± 0.28 Mb; Wilcoxon’s paired test, *p*-value < 0.05; Supplementary Figure 11) in BGC genome percent between genomes from both environments.

For the 159-subset, the correlation between the number of BGC-dedicated base pairs per genome and genome size was higher (0.821 rho Spearman’s correlation, *p*-value < 0.05) than that predicted by Shih et al. (2013) (*R*^2^ = 0.3, *p*-value < 0.0001), but similar for the hot spring and non-thermal genomes (0.799 and 0.761, respectively). The latter suggests that the streamlining of hot spring genomes also involves a reduction in BGCs. An ecological explanation for BGC-loss in hot spring genomes could be the lower diversity of these microbial communities compared to other environments (Li et al., 2014). In hot spring communities, the role of secondary metabolites as weapons of inter-microbial warfare (O’Brien and Wright, 2011) could be diminished and susceptible to loss. Furthermore, viral and exogenous nucleic acids are less diverse in hot springs (Parmar et al., 2018), and cyanobacteria are subjected to strong viral predation and potential coevolution (Guajardo-Leiva et al., 2018), as also seen for other thermophilic organisms (Breitbart et al., 2004). Therefore, the prevalence of the CRISPR-Cas system in their genomes could be more important than in non-thermal genomes as seen in other phyla (Weinberger et al., 2012).

The ability to survive at higher temperatures has been gained and lost across bacteria and archaea (Pollo et al., 2015), and all genomic features common to thermophiles are not mandatory for all high-temperature organisms (Puigbò et al., 2008). This was also observed in this study, suggesting once again that different adaptation strategies exist. The polyphyly of this feature in cyanobacteria, seen here widespread in almost all orders, is explained as secondary adaptation (not basal) during the niche expansion stage of trait evolution for this phylum and is intimately related with the ability to form microbial mats (Hammerschmidt et al., 2020). Furthermore, the number of transitions to and from thermophily in cyanobacteria has shown a strong reduction between 0.9 and 0.8 Gya (during the cold temperatures of the Neoproterozoic Oxidation Event), and a slight increase in transitions over the last 0.3 Gya (warmer temperatures). The growing numbers of genera with both environmental groups here described increase the evidence of these recent transitions to thermophily. Altogether, it demonstrates the effect of global temperature changes during this niche expansion (Uyeda et al., 2016) and in the worldwide dispersal of thermophilic cyanobacteria, like *Fischerella*, during the global rise in air temperature 74 mya (Miller et al., 2007). Studying the pathways by which different groups of hot spring cyanobacteria became the autotrophic base for microbial mats in these environments will be the foundation for understanding how different organisms cope and proliferate at higher temperatures in a currently changing world.

## Conclusion

Cyanobacteria are essential primary producers in hot spring phototrophic microbial mats. In this study, metagenomic binning was used to obtain 57 new cyanobacterial MAGs, subsequently revealing a wide distribution of new thermophilic cyanobacterial members across the phylum. How these cyanobacteria began to colonize these environments is still unknown; however, the adaptation to high temperatures have strong genomic consequences for cyanobacteria that currently live in hot springs. The transition from a non-thermal environment to hot springs is reflected in genomic properties that could be more advantageous to survival, such as small genomes and warm-adapted proteins, as well as a higher abundance of specific protein functional categories to cope with mineral water composition and new microbial and viral communities. This study demonstrates how adaptation to hot spring environments can be traced at the genus level, showing that recent divergences could be restricted to a small set of genes that can be shared by diverse members within the same niche. Under the current scenario of global change, it is important to understand the evolution of hot spring genomes as an example of selective pressure by warmer environments. Studying the information within MAGs from lower hot spring temperatures will allow us to predict changes in genomic features that many species may face in both present and future scenarios on Earth.

## Data Availability Statement

The datasets presented in this study can be found in online repositories. The names of the repository/repositories and accession number(s) can be found below: https://www.ncbi.nlm.nih.gov/, (NCBI BioProject numbers PRJNA635751 and PRJNA645256); https://figshare.com/, 10.6084/m9.figshare.1240097.

## Author Contributions

JA and BD conceived the study. JA, OS, and TA-S analyzed the metagenomic database and obtained the metagenome-assembled genomes. JA and TA-S performed the evolutionary and bioinformatic analyses under supervision of BD. JA and BD wrote the manuscript. All authors read, commented and approved the drafted manuscript.

## Conflict of Interest

The authors declare that the research was conducted in the absence of any commercial or financial relationships that could be construed as a potential conflict of interest.

## Footnotes

1 http://hmmer.org/

2 https://data.ace.uq.edu.au/public/misc_downloads/refinem/

3 https://github.com/tseemann/barrnap

4 https://github.com/dparks1134/CompareM

5 https://knowledge.figshare.com/

6 http://www.insdc.org/tpa.html

7 http://bbtools.jgi.doe.gov
