# Supplementary material for: Taxonomic Novelty and Distinctive Genomic Features of Hot Spring Cyanobacteria
Source: Front Genet. 2020 Nov 5;11:568223. doi: 10.3389/fgene.2020.568223 (PMC7674949; doi:10.3389/fgene.2020.568223)
Supplement: Supplementary file 1 [file Data_Sheet_1.PDF]

Supplementary figures

Figure S1. Location of non-thermal genomes in PCC-7336 and Thermosynechococcales orders.

Figure S2. Ecological relationships of cyanobacterial abundances.

Figure S3. Distribution of thermophilic cyanobacterial MAGs.

Figure S4. Comparison of expected genome size between cyanobacterial genomes.

Figure S5. Comparison of GC content between cyanobacterial genomes.

Figure S6. Comparison of coding density between cyanobacterial genomes.

Figure S7. Comparison of amino acid frequencies between cyanobacterial genomes.

Figure S8. Exclusive orthogroups for various taxa from hot spring or non-thermal genomes.

Figure S9. Phylogeny for orthogroups distributed in hot spring genomes from various taxa.

Figure S10. Patterns of COG distribution in core and accessory orthogroups.

Figure S11. Comparison of genomic percentage corresponding to BGCs between cyanobacterial genomes.

Supplementary Table captions

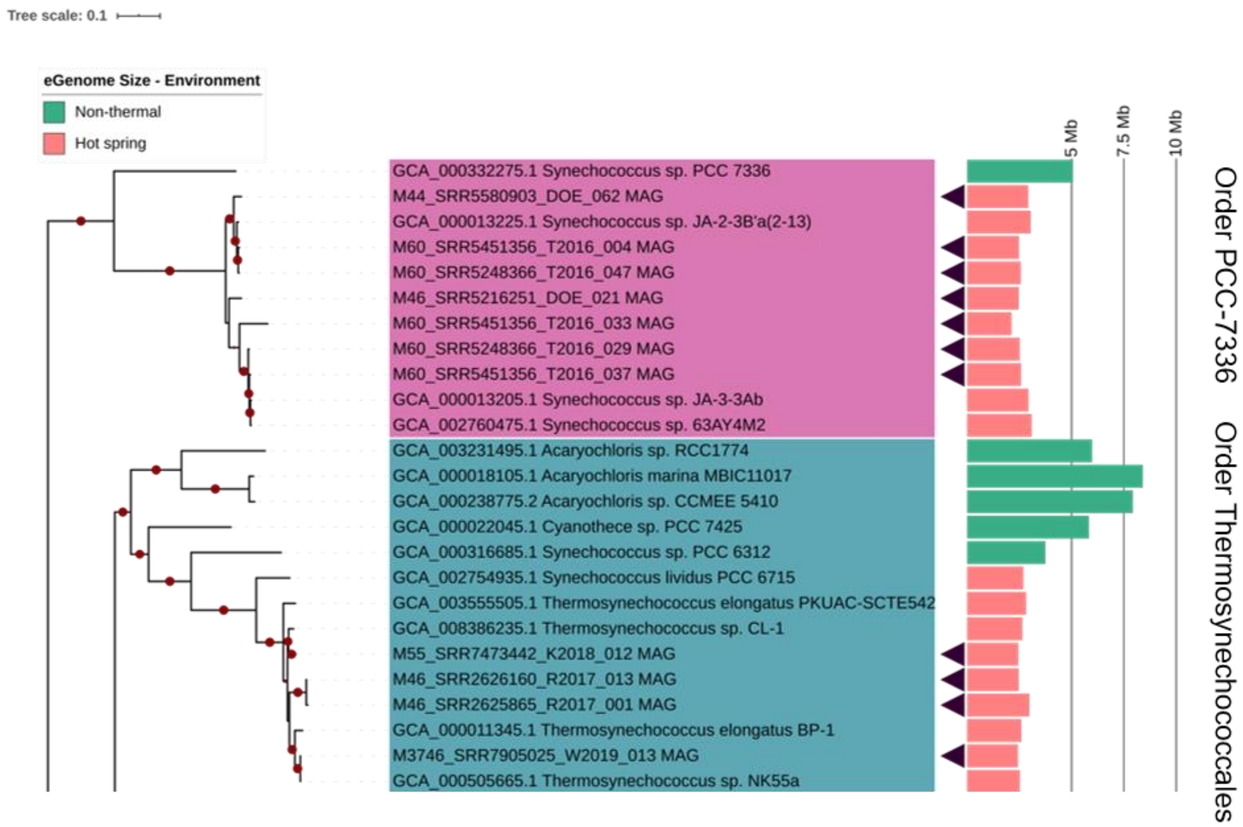

Figure S1. **Location of non-thermal genomes in PCC-7336 and Thermosynechococcales orders.** Pruned tree from Figure 4 showing only the genomes corresponding to PCC-7336 and Thermosynechococcales orders. Maximum likelihood tree reconstruction was done with IQtree software using LG+R10 model and a non-parametric UF-bootstrap support of 1000 replicates. Red squares in tree represent UFbootstrap support > 90 % for the respective node. Bars are proportional to the expected genome size between the genomes of the order being red the ones from hot springs and green the non-thermal. Purple arrows represent cyanobacterial MAGs recovered in this study.

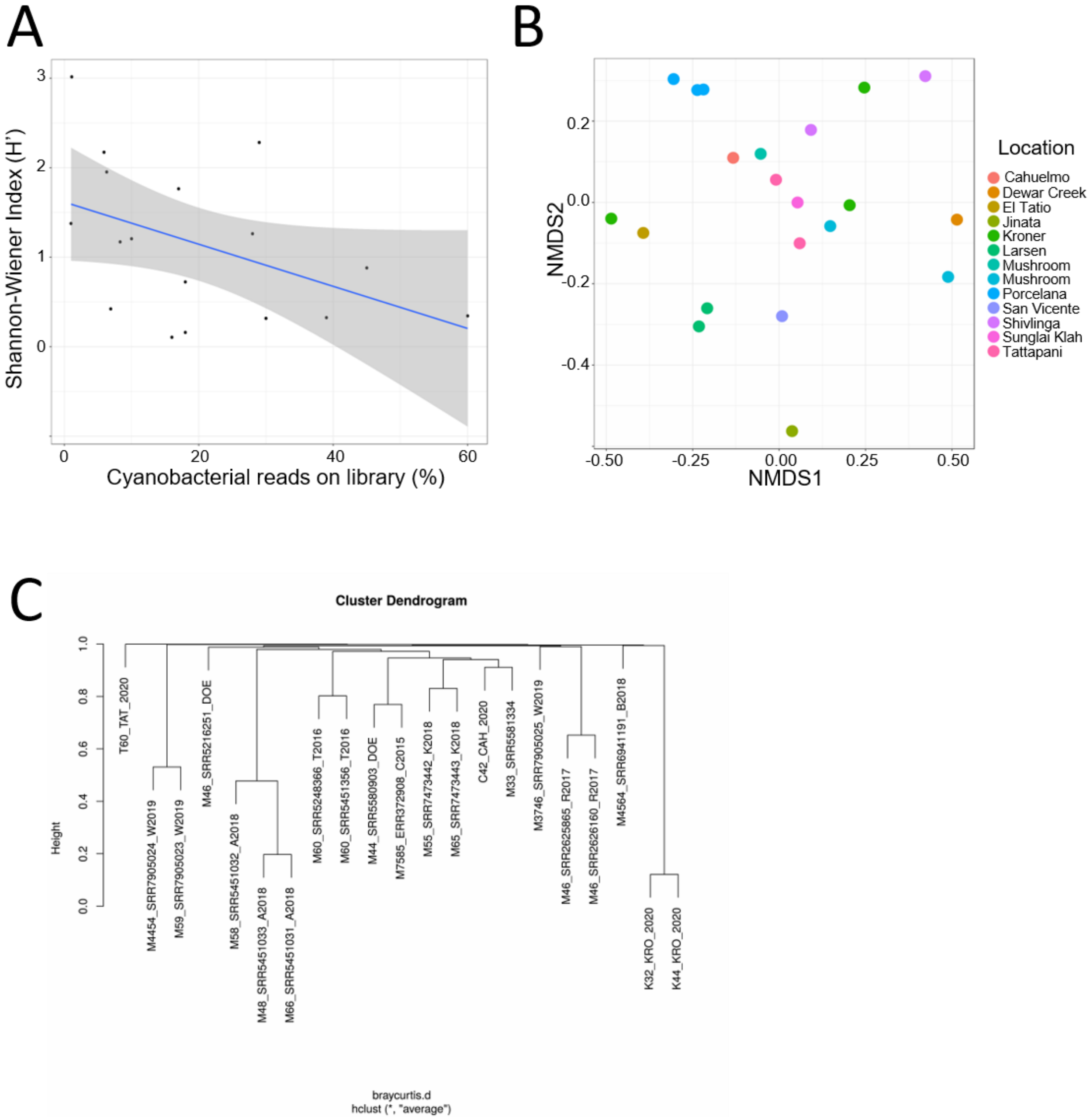

Figure S2. **Ecological relationships of cyanobacterial abundance**s. A) Correlation between alpha-diversity and normalized abundances of cyanobacterial reads of each metagenome. B) Non-metric multidimensional scaling (NMDS) based on the absolute abundances of MAGs in their respective

metagenomes. C) Bray curtis dissimilarity clustering between all samples based on their cyanobacterial composition. Metagenome names and locations are listed in Table S1.

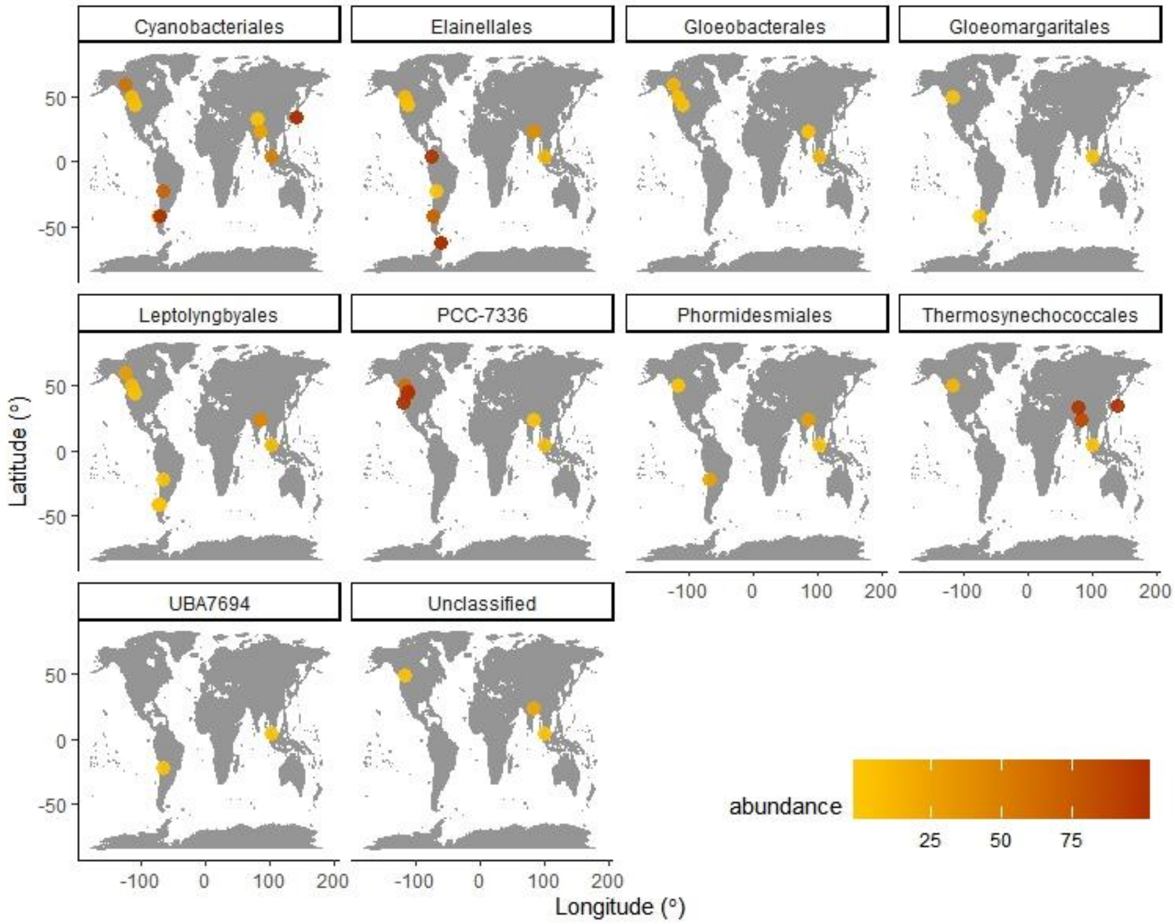

Figure S3. **Distribution of thermophilic cyanobacterial MAGs.** Maps of the distribution and relative normalized abundances of cyanobacterial MAGs at order level in the different metagenomes. Scale bar represents the normalized abundances as cyanobacterial reads percentages of each order and metagenome. The possible new order M55_SRR7473442_K2018_030 corresponds to the Unclassified category.

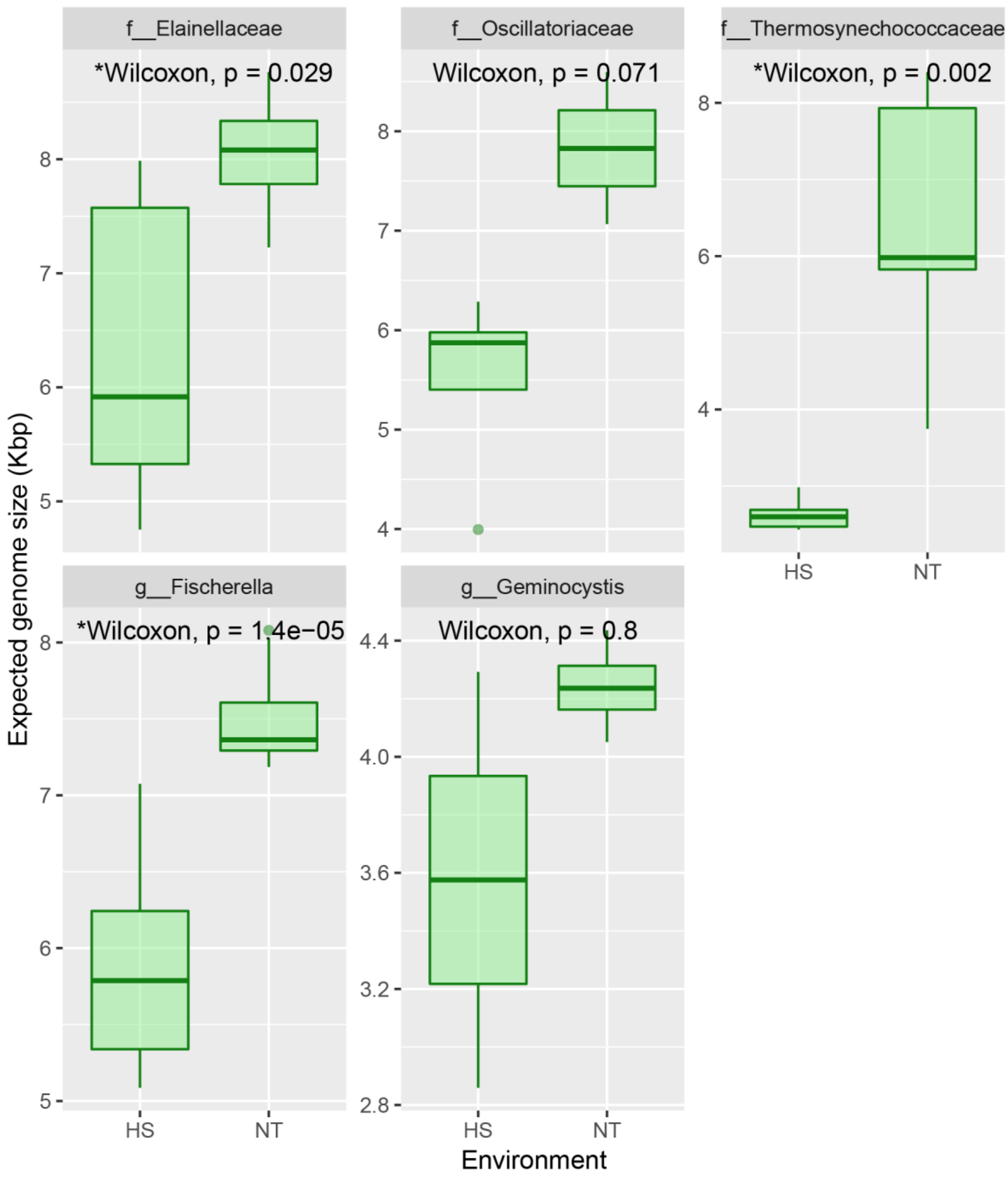

Figure S4. **Comparison of expected genome size between cyanobacterial genomes.** Expected genome size was determined as a correction of observed genome size by the completeness of the genomes (see methods). Comparison of expected genome size was done for hot spring (HS) and non-

thermal (NT) genomes inside the same family or genus with ≥3 genomes for each environmental group. Wilcoxon's paired test p-values with FDR correction are shown inside the box plots.

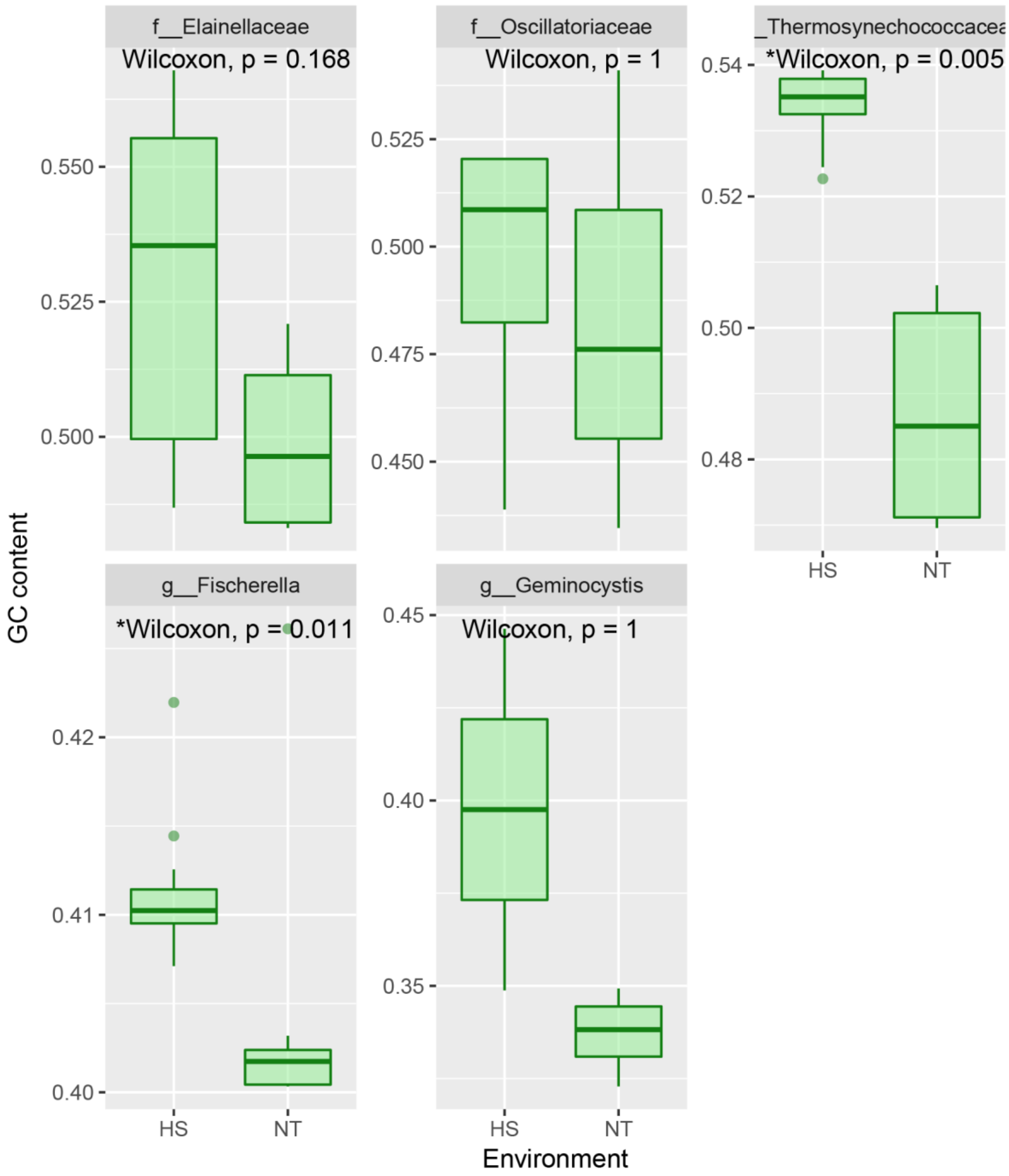

Figure S5. **Comparison of GC content between cyanobacterial genomes.** Comparison of GC content was done for hot spring (HS) and non-thermal (NT) genomes inside the same family or genus with ≥3 genomes for each environmental group. Wilcoxon's paired test p-values with FDR correction are shown inside the box plots.

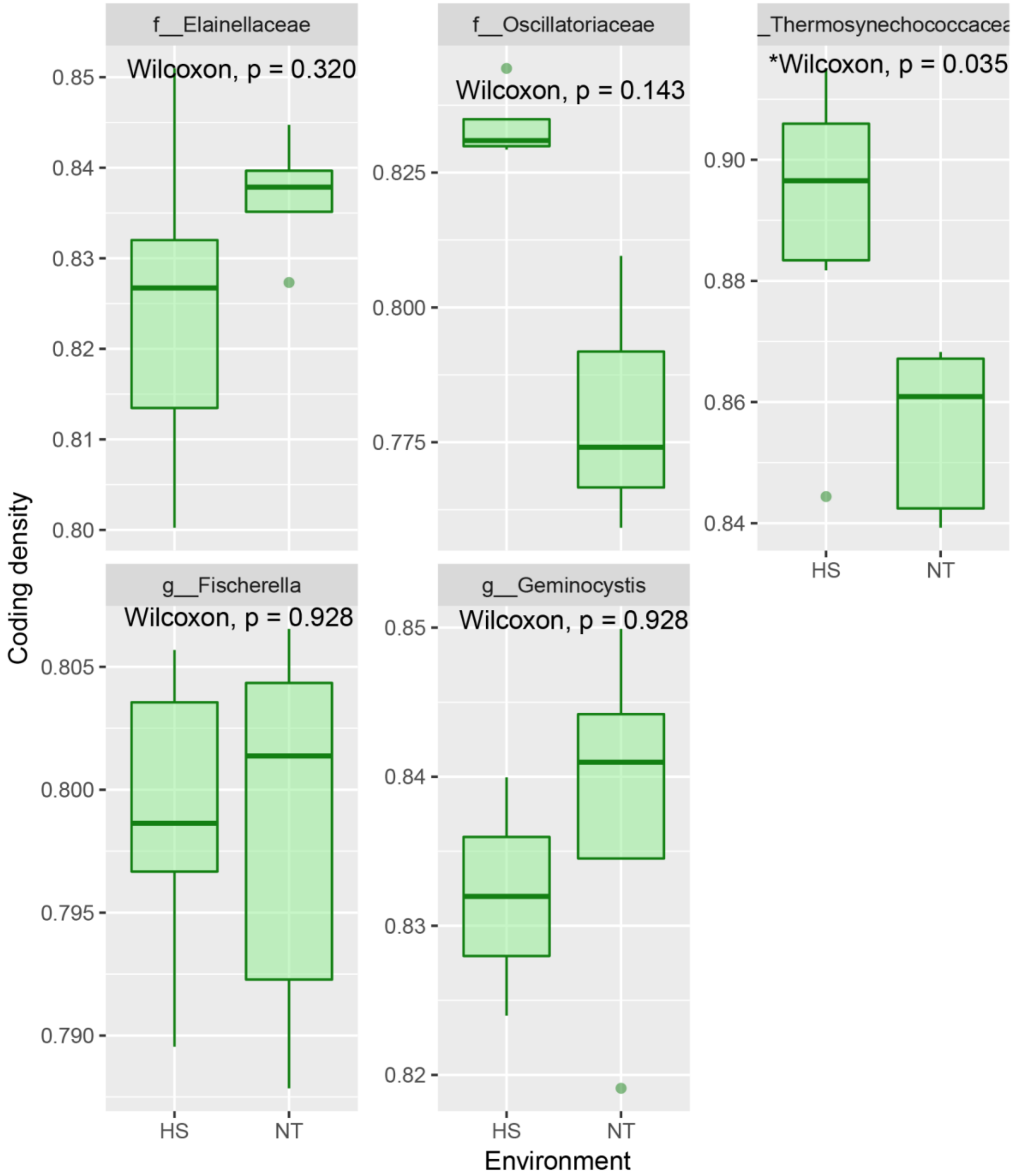

Figure S6. **Comparison of coding density between cyanobacterial genomes.** Comparison of coding density was done for hot spring (HS) and non-thermal (NT) genomes inside the same family or genus with ≥3 genomes for each environmental group. Wilcoxon's paired test p-values with FDR correction are shown inside the box plots.

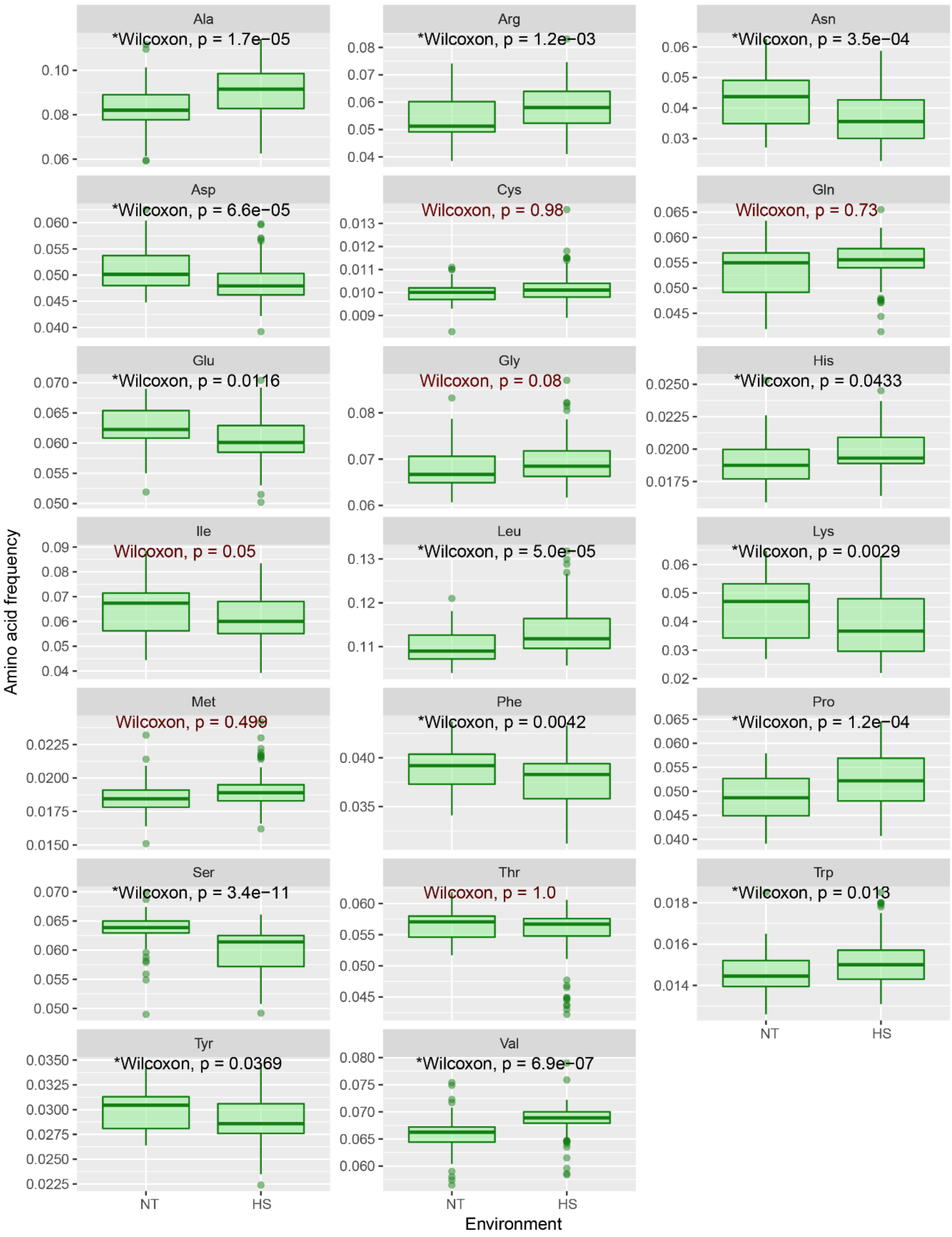

Figure S7. **Comparison of amino acid frequencies between cyanobacterial genomes.** Comparison of amino acid frequency was done for the 159-subset of hot spring (HS) and non-thermal (NT) genomes. Wilcoxon's paired test p-values with Bonferroni correction are shown inside the box plots. Corrected p-values > 0.05 are colored in red.

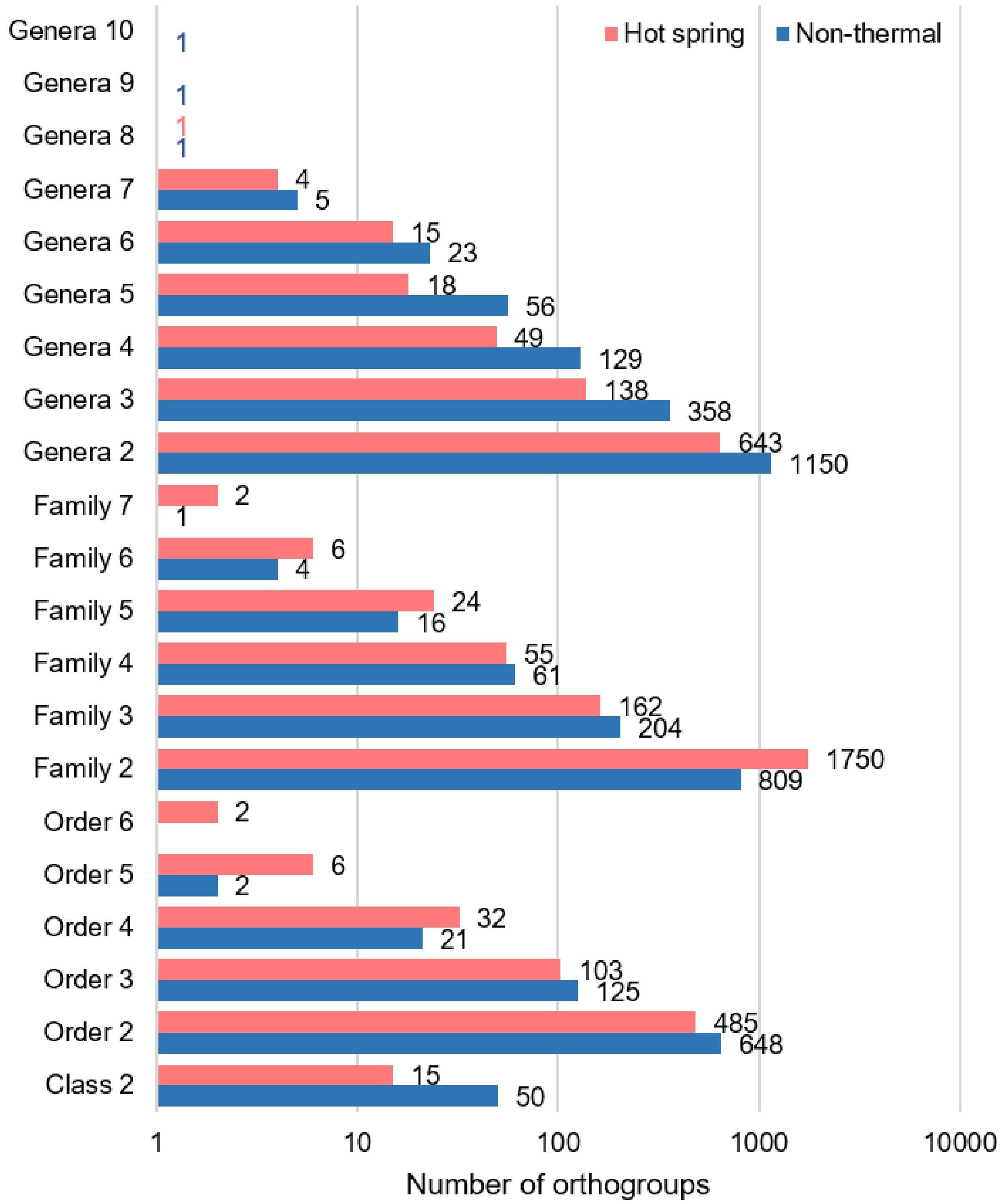

Figure S8. **Exclusive orthogroups for various taxa from hot spring or non-thermal genomes.** Taxonomic distribution of orthogroups that are exclusive to each environmental group and shared between two or more clades at each taxonomic rank. The raw values are shown at the right side of each bar plot. The x-axis is represented as the number of orthogroups in la log_{10} scale.

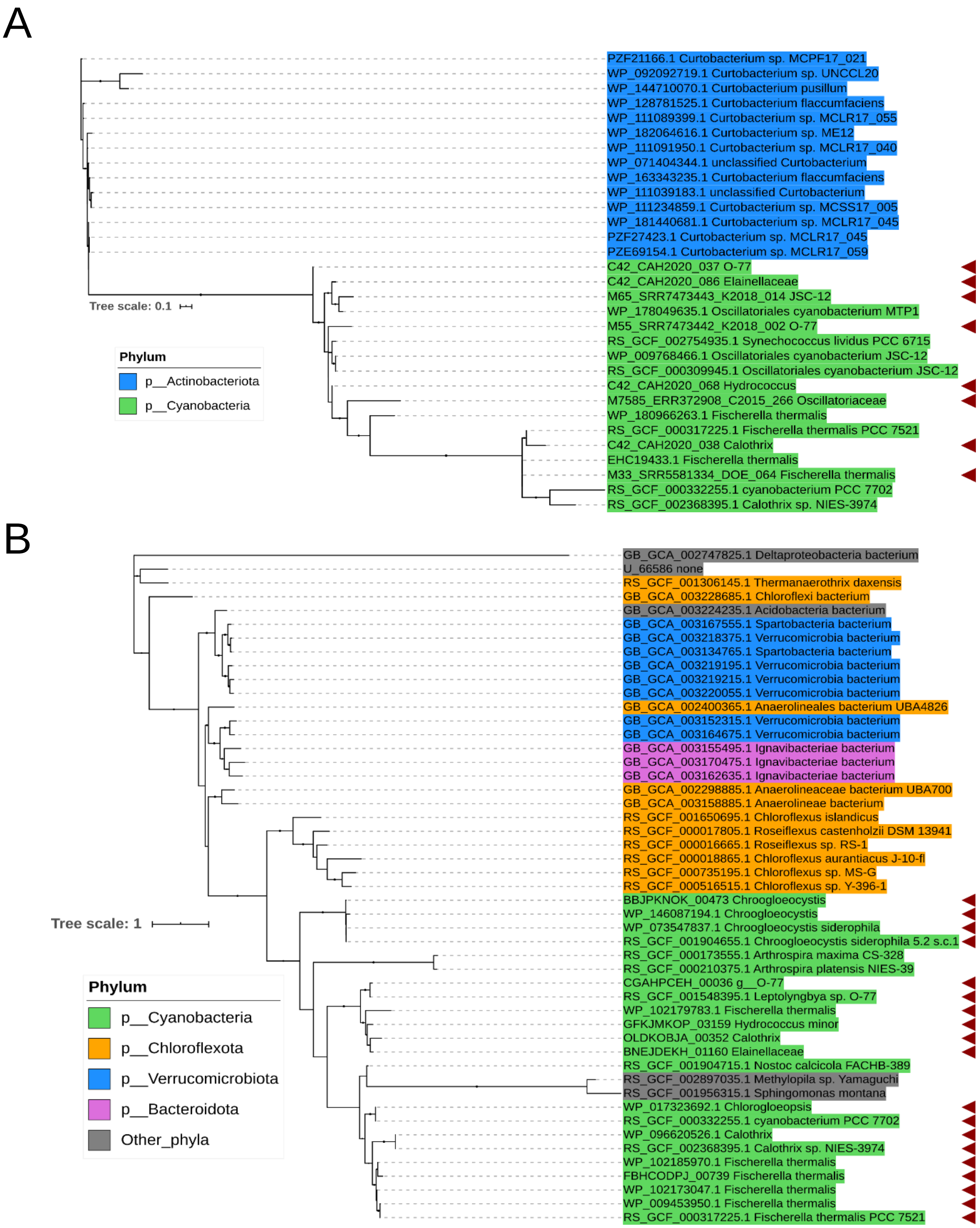

Figure S9. **Phylogeny for orthogroups distributed in hot spring genomes from various taxa.** Maximum likelihood tree reconstruction for hypothetical protein orthogroups A) OG0008066 (shared between 5 families) and B) OG0006223 (shared between 7 genera). Reconstruction was done with IQtree software using JTT+F+R2 (A) and LG+F+G4 (B) models and a non-parametric UF-bootstrap support of 1000 replicates. Red arrows represent cyanobacterial MAGs recovered in this study.

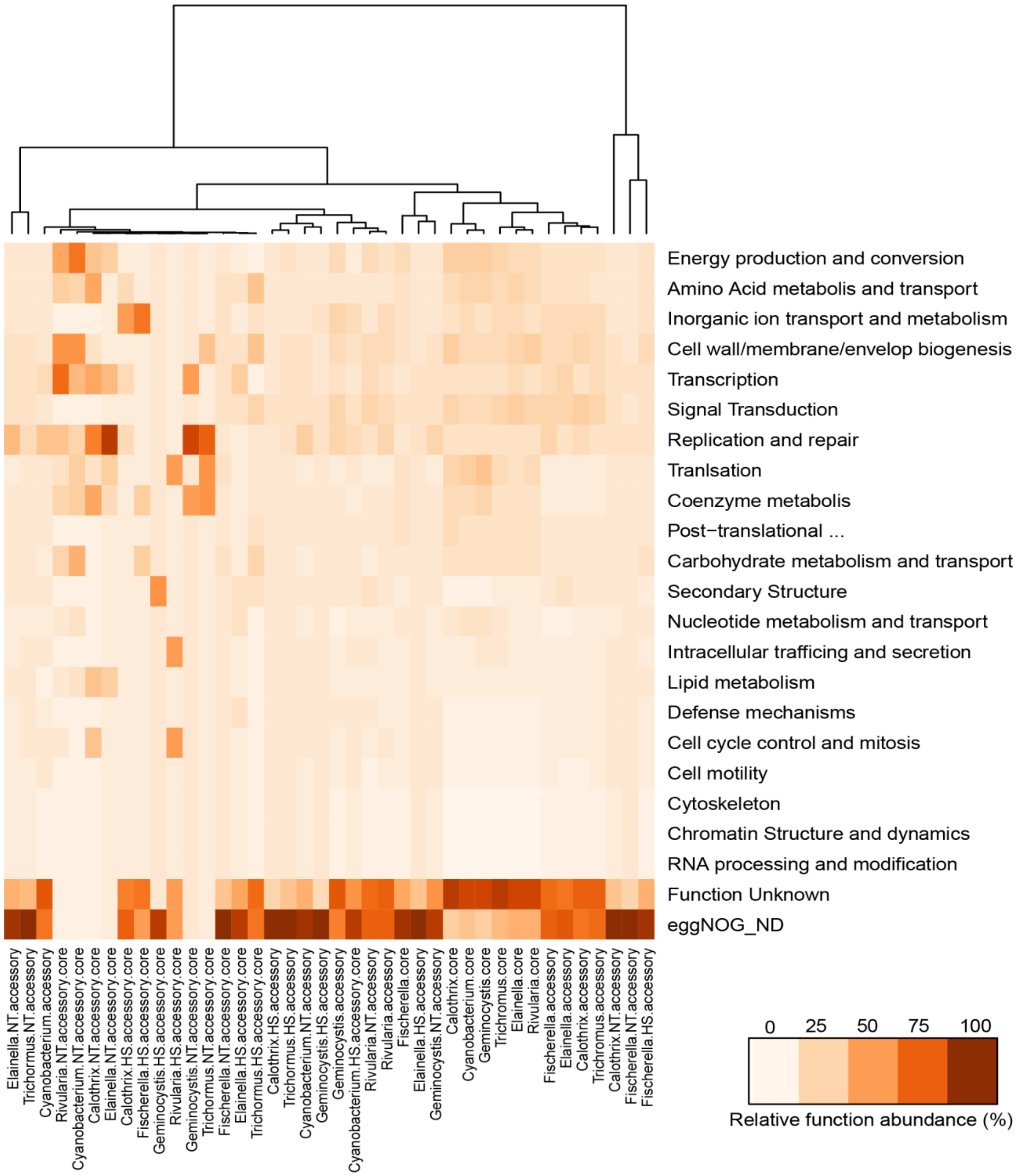

Figure S10. **Patterns of COG distribution of core and accessory orthogroups.** Heatmap representing the percentage of COG functions in the set of orthogroups classified as core, accessory core (in all genomes for the genus) for non-thermal (NT) or hot spring (HS) genomes, accessory for NT or HS (not in all genomes of the genus) and accessory for the genus (not specific for any environmental group). Analyses were done for the following genera *Calothrix*, *Cyanobacterium*, *Elainella*, *Fischerella*, *Geminocystis*, *Rivularia* and *Trichormus*. Hierarchical clustering was done according to x-axis and relative function abundances were calculated for each column.

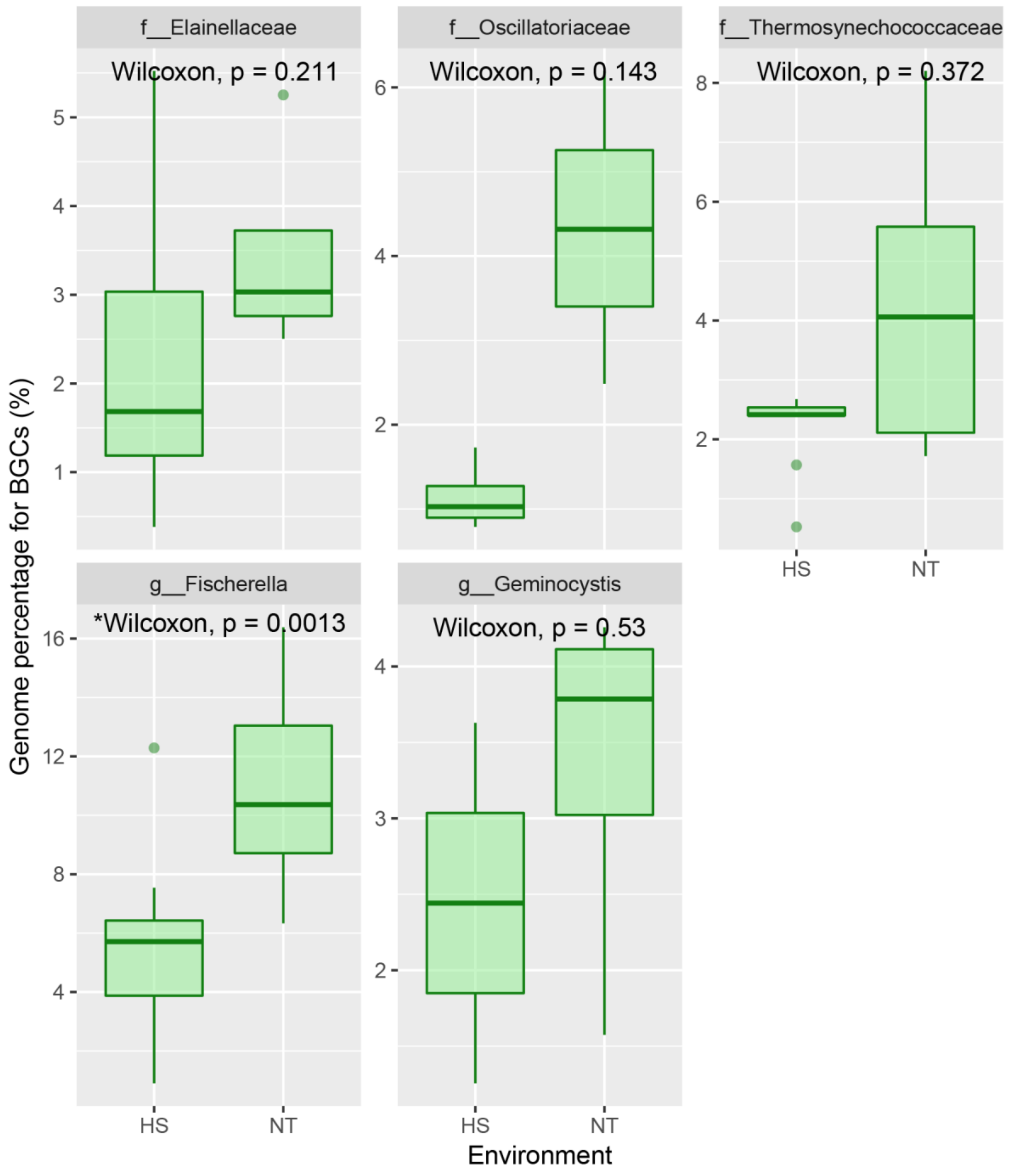

Figure S11. **Comparison of genomic percentage corresponding to secondary metabolite gene clusters between cyanobacterial genomes.** Comparison of the genomic percentages of BGCs was done for hot spring (HS) and non-thermal (NT) genomes inside the same family or genus with ≥3 genomes for each environmental group. Wilcoxon's paired test p-values with FDR correction are shown inside the box plots.

Supplementary Table captions

Table S1. **Metagenomes used in this study**. The metagenomes used in this study are ordered according to the prefix given for their respective MAGs. Temperature and pH were retrieved from the respective NCBI BioSample or directly from the associated reference. Numbers of total MAGs and cyanobacterial MAGs obtained are given. The detailed information for each SRA metagenome was retrieved directly from the NCBI database.

Table S2. **Taxonomy and information of the 857 genomes used in this study**. Each genome was analyzed with the checkM software and the obtained features are showed. The GTDB-tk analyses are also showed. For the 57 cyanobacterial MAGs the tRNAs and presence of 16S, 5S and 23S rRNA subunits (partial or complete) are also showed.

Table S3. **Genome information for the 159-subset**. The NCBI names, environments and references were obtained from the nucleotide NCBI database for each genome. The retrieved Environments_2 were classified in wider categories (Environment_1) and according to this were classified into Hot spring (1) or non-thermal genomes (0). GTDB classification and expected genome size were obtained from Table S2.

Table S4. **Orthogroups of the 159-subset.** The orthogroups obtained from the Orthofinder software for the 159-subset of genomes are showed, indicating if the genomes were classified as hot spring or non-thermal and the orthogroups if they were core, hot spring accessory, non-thermal accessory, phylum accessory or singletons.

Table S5. **Classification of all MAGs obtained from the 21 metagenomes.** Phylum level classification of the 1152 MAGs obtained from the 21 metagenomes used in this study. The classification was obtained with the GTDB-tk software and the percentage that represent each phyla regarding all MAGs is shown.

Table S6. **Orthogroups shared across hot spring cyanobacterial taxa.** Orthogroups from only hot spring genomes (accessory) that were shared over 4 genera, 4 families or 2 orders are listed with the respective accession numbers and annotations, consensus annotation and ordered according to the highest number and highest taxonomic level in which were present.

Table S7. **Orthogroups of the seven genera with hot spring and non-thermal genomes.** The orthogroups obtained from the Orthofinder software for each genera (*Calothrix*, *Cyanobacterium*, *Elainella*, *Fischerella*, *Geminocystis*, *Rivularia* and *Trichormus*) are showed, indicating if the genomes were classified as hot spring or non-thermal and the orthogroups if they were core, hot spring core accessory, hot spring accessory, non-thermal core accessory, non-thermal accessory and genus accessory. Singletons are included in the specific non-core accessory groups.
